# Palmitic acid coordinates impaired visceral adipose ICOS^hi^ Treg-mediated immunosuppression and systemic metabolic disturbance during obesity

**DOI:** 10.1172/JCI207089

**Published:** 2026-07-23

**Authors:** Weitong Su, Yuxiao Liu, Xi Yan, Mengyao Huang, Linghao Xu, Jing Lin, Xufeng Chen, Puyuan Hu, Chenlin Gao, Jian Wen, Hongdong Wang, Dong Ding, Zengpeng Zheng, Wenjing Li, Lianjia Li, Zhan Liu, Keyu Qian, Jing Gao, Tingting Zhang, Xiaobing Mao, Haibing Zhang, Wei Lu, Bin Li, Hong Li, Aoyuan Cui, Yan Bi, Chunxiang Zhang, Yu Li

**Affiliations:** 1 Shanghai Institute of Nutrition and Health, University of Chinese Academy of Sciences, Chinese Academy of Sciences, Shanghai, China.; 2 Department of Endocrinology and Metabolism, Metabolic Vascular Disease Key Laboratory of Sichuan Province, The Affiliated Hospital of Southwest Medical University, Luzhou, Sichuan, China.; 3 Department of General Surgery (Hepatobiliary Surgery), Biliary-Pancreatic Center, The Affiliated Hospital, Southwest Medical University, Luzhou, Sichuan, China.; 4 Metabolic Hepatobiliary and Pancreatic Diseases Key Laboratory of Luzhou City, Academician (Expert) Workstation of Sichuan Province, The Affiliated Hospital, Southwest Medical University, Luzhou, Sichuan, China.; 5 Department of Endocrinology, Endocrine and Metabolic Disease Medical Center, Nanjing Drum Tower Hospital, Affiliated Hospital of Medical School, Nanjing University, Nanjing, China.; 6 Department of Immunology and Microbiology, Shanghai Institute of Immunology, Shanghai Jiao Tong University School of Medicine, Shanghai, China.; 7 Metabolic Vascular Disease Key Laboratory of Sichuan Province, The Affiliated Hospital of Southwest Medical University, Key Laboratory of Medical Electrophysiology, Ministry of Education, Southwest Medical University, Luzhou, China.

**Keywords:** Inflammation, Metabolism, Obesity, T cells

## Abstract

Tregs in visceral adipose tissue (VAT) play essential roles in systemic metabolic homeostasis under distinct physiological and pathological conditions. However, the metabolic cues that drive Treg subset specialization in the obese VAT niche remain elusive. Here, we demonstrated that palmitic acid (PA) instigated chronic VAT inflammation and systemic metabolic disturbance by compromising the immunosuppressive function of the ICOS^hi^ Treg subset. PA, but not oleic acid, activated CREB/ATF bZIP transcription factor (*Crebzf*) expression in VAT Tregs from high-fat, high-sucrose diet–induced (HFHS diet–induced) obese and ob/ob mice. *Crebzf* deficiency significantly attenuated diet-induced obesity and inflammation by upregulating the suppressive function of VAT ICOS^hi^ Tregs. Moreover, adoptive transfer of *Crebzf*-deficient ICOS^hi^ Tregs into *Rag*1*^–/–^* mice alleviated HFHS diet–induced inflammation and metabolic disorders more effectively than transfer of *Crebzf*-sufficient ICOS^hi^ Tregs. Mechanistically, CREBZF interacted with c-JUN to inhibit *Foxp3* activity, thereby impairing the stability and inhibitory cytokine production of ICOS^hi^ Tregs. In humans, *CREBZF* levels in VAT Tregs were elevated and negatively correlated with *FOXP3* activity. Collectively, these findings uncover a specific ICOS^hi^ Treg subset that responds to PA, thereby coupling obesogenic signals to VAT remodeling and systemic metabolic homeostasis.

## Abstract 2

Palmitic acid impairs a specific VAT Treg cell subset, fueling obesity-driven inflammation. Restoring this subset’s function may combat systemic metabolic disturbance.

## Graphical abstract

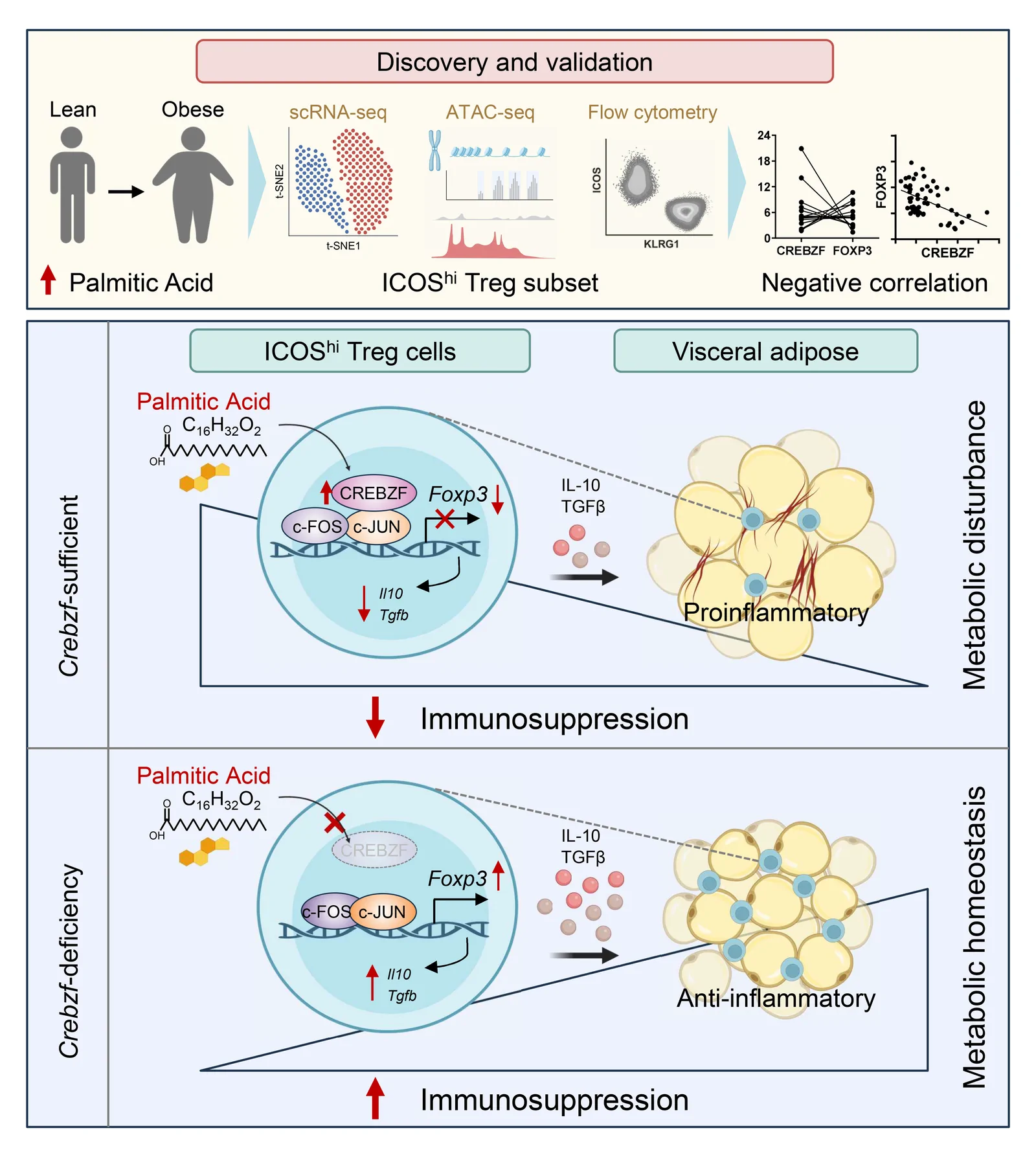

## Introduction

Obesity develops with disturbed energy balance and is accompanied by systemic low-grade inflammation and contributes to metabolic disorders, such as insulin resistance, type 2 diabetes mellitus (T2DM), and cardiovascular diseases (1–4). The chronic proinflammatory state is a hallmark of visceral white adipose tissue (VAT) dysfunction and systemic metabolic disturbance (5). Previous studies have demonstrated the importance of T cell subsets in regulating inflammatory status and functional remodeling of VAT in response to metabolic cues during obesity (6–8). Therefore, new insights into T cell subset–mediated regulation of adipose tissue may reveal strategies for treating obesity and metabolism-related diseases.

Tregs serve essential roles in immune tolerance and systemic tissue homeostasis (9, 10). The transcription factor FOXP3 serves as the master regulator that defines Treg lineage and is essential for maintaining its functional stability and suppressive capacity (11–13). In addition, T cell receptor (TCR) repertoires along with growth and survival factor dependencies support Treg fate and tissue-resident functions (14, 15). A specialized population of CD4^+^ FOXP3^+^ regulatory T cells residing in visceral adipose tissue (VAT) accumulates in lean individuals and plays a beneficial role in maintaining systemic glucose metabolism and insulin sensitivity (6, 8, 16). The VAT Treg compartment has recently been recognized as a highly heterogeneous cell population that responds to specific microenvironmental signals (17–19). Insulin signaling facilitates the transition from CD73^hi^ST2^lo^ to CD73^lo^ST2^hi^ Tregs to limit beige adipogenesis (17). In parallel, sex hormone signals govern the functions of distinct VAT Treg subsets to modulate glucose homeostasis and restrain inflammation (18, 20). Although current understanding of VAT Treg responses derives primarily from various physiological contexts, the heterogeneity and pathological adaptation of VAT Tregs within the obesity environment remain largely unexplored.

Given that nutritional cues and hormone signals are critical for the regulation and suppressive function of VAT Tregs (21–24), and given the elevated levels of saturated fatty acids in the obese milieu, whether and how saturated fatty acids are sensed by Tregs and subsequently affect adipose tissue homeostasis remains to be addressed. We have previously identified the CREB/ATF bZIP transcription factor (CREBZF) as a pivotal regulator and established its functional importance in hepatic steatosis, liver regeneration, and metabolic dysfunction–associated steatohepatitis (MASH) progression (25–27). Furthermore, our recent studies demonstrated that CREBZF in adipocytes regulates thermogenesis, energy homeostasis, and inflammation (28, 29). However, whether CREBZF could exert immunometabolic regulatory effects through specific T cell subsets remains to be determined.

Here, we identify what we believe to be a previously unrecognized function of CREBZF in mediating the sensing of palmitic acid (PA) to specifically limit the stability and suppressive function of a protective ICOS^hi^ Treg subset, thereby exacerbating VAT inflammation and systemic metabolic disturbance. This study demonstrates that ICOS^hi^ Tregs constituted a specialized subset within the obese visceral adipose niche. Notably, PA disrupted the stability and suppressive function of VAT ICOS^hi^ Tregs. CREBZF deficiency enhanced visceral adipose tissue ICOS^hi^ Treg‑mediated immunosuppression and reduced metabolic disturbance during obesity. Finally, we found that the stability and suppressive function of VAT Tregs were negatively correlated with CREBZF levels in both obese mice and humans.

## Results

### Elevated CREBZF in visceral adipose–resident T cells from humans and mice with obesity.

**Figure 1:**
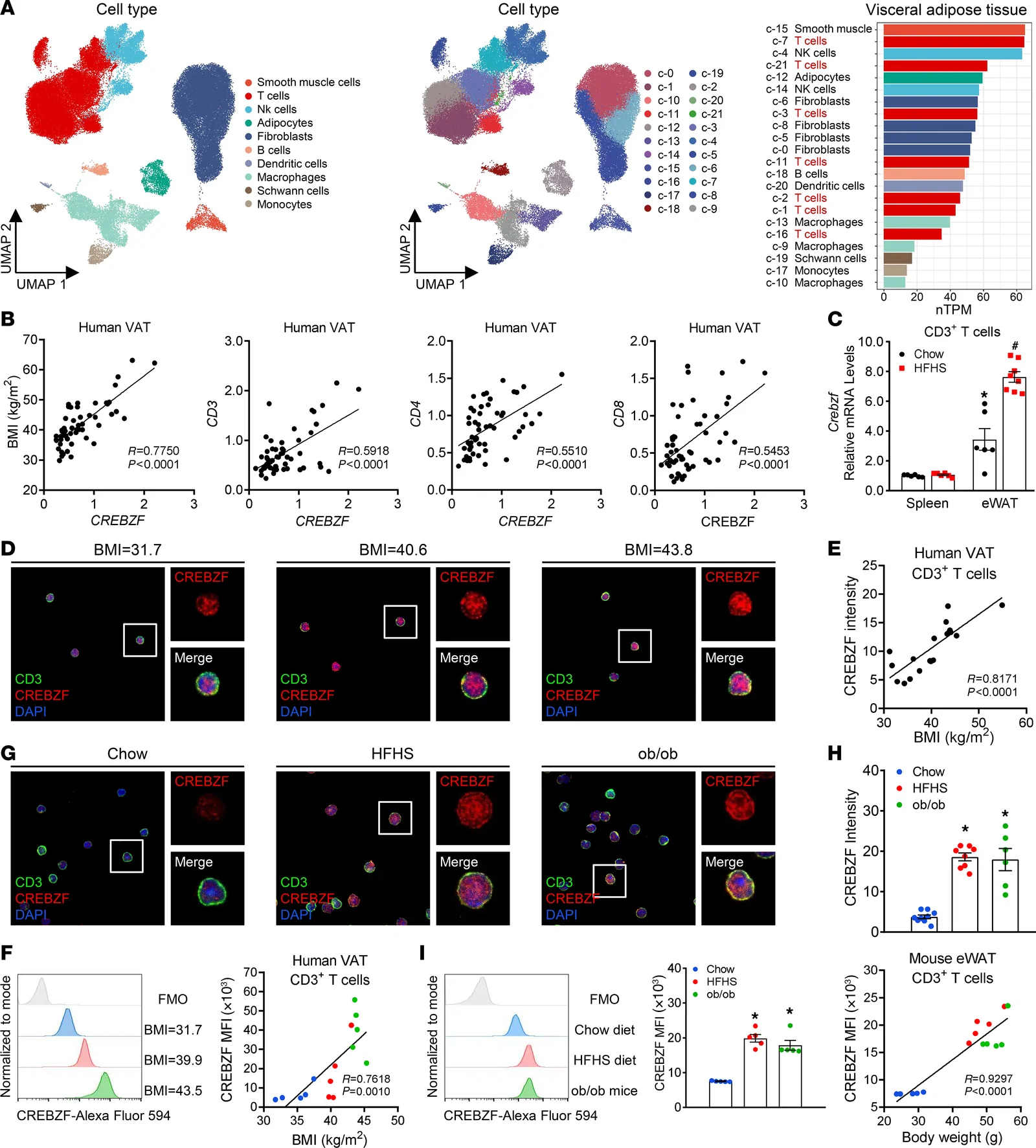
CREBZF is elevated in adipose tissue T cells in humans and mice with obesity. (**A**) *CREBZF* expression in different cell types (left) and clusters (middle) identified in human adipose tissue and visualized by a UMAP plot, as well as a bar chart (right). The T cell clusters are highlighted in red. (**B**) Pearson correlation of *CREBZF* mRNA levels with BMI and Tconv cell marker genes in human VAT. *n* = 53–55. (**C**) The mRNA levels of *Crebzf* in T cells isolated from spleens and eWAT of mice fed a chow or HFHS diet. Data are presented as the mean ± SEM. *n* = 6–8. Two-way ANOVA for multiple comparison. *P < 0.05, versus spleen and chow; ^#^*P* < 0.05, versus eWAT and chow. (**D**–**F**) CREBZF expression in human VAT T cells positively correlated with BMI. (**D**) Representative immunofluorescence images of CD3^+^ T cells from VAT from individuals with varying BMIs. Original magnification, ×630. Scale bars: 25 µm (square side length). (**E**) Correlation between CREBZF intensity of CD3^+^ T cells and BMI. (**F**) Representative histograms of CREBZF in CD3^+^ T cells from human VAT and the correlation of CREBZF MFI with BMI. (**G** and **H**) The expression of CREBZF is increased in eWAT T cells from obese mice. (**G**) Representative immunofluorescence images of CREBZF in CD3^+^ T cells from eWAT of chow-fed, HFHS diet–fed, or ob/ob mice. Original magnification, ×630. Scale bars: 25 µm (square side length). (**H**) Quantification of CREBZF intensity from **G**. *n* = 6–9. *P < 0.05 versus chow diet–fed mice. (**I**) Representative histograms (left) and MFI (middle) of CREBZF in eWAT CD3^+^ T cells from chow diet–fed, HFHS diet–fed, or ob/ob mice, and the correlation (right) between CREBZF MFI and mouse body weight. Data are presented as the mean ± SEM. *n* = 5. One-way ANOVA for multiple comparison. *P < 0.05 versus chow diet–fed mice.

Single-cell transcriptome data from the Human Protein Atlas (HPA) shows that CREBZF, a key regulator in metabolic homeostasis (25, 30, 31), is highly expressed in T cells compared with other immune cell types in VAT and the vasculature (Figure 1A and Supplemental Figure 1A; supplemental material available online with this article; https://doi.org/10.1172/JCI207089DS1). High levels of CREBZF expression are observed in both human and murine immune tissues, with even higher levels in T cells (Supplemental Figure 1, B–D). In VAT from individuals with obesity, *CREBZF* mRNA expression was positively associated with BMI and the expression of T cell markers, including the pan–T cell marker *CD3*, the helper T cell marker *CD4*, and the cytotoxic T cell marker *CD8* (Figure 1B). In addition, *CREBZF* levels correlated with metabolic parameters of insulin resistance, specifically the homeostasis model assessment of insulin resistance (HOMA-IR) and plasma insulin levels (Supplemental Figure 1E). However, *CREBZF* expression in subcutaneous adipose tissue (SAT) showed a slight correlation with these T cell markers (Supplemental Figure 1F). Furthermore, we confirm that CD3^+^ T cells isolated from epididymal white adipose tissue (eWAT) of high-fat, high-sucrose diet–fed (HFHS diet–fed) mice expressed higher levels of CREBZF than did T cells from the spleen or chow diet–fed mice (Figure 1C). These findings indicate a previously unappreciated cell-intrinsic role for CREBZF, specifically in T cells within VAT.

To further determine CREBZF expression in T cells from VAT in obesity, we performed immunofluorescence staining and flow cytometry. Interestingly, CREBZF expression in VAT-derived CD3^+^ T cells increased proportionally to BMI in obese individuals, exhibiting a significant positive correlation with BMI (Figure 1, D–F, and Supplemental Figure 1, G and H). Consistently, CREBZF levels were dramatically elevated in CD3^+^ T cells isolated from eWAT of both HFHS diet–fed and ob/ob mice, strongly correlating with mouse body weight (Figure 1, G–I). Taken together, these results demonstrate that CREBZF expression was elevated in CD3^+^ T cells from VAT and eWAT of humans and mice with obesity, suggesting that CREBZF may contribute to altered T cell function and immune homeostasis in the adipose niche during obesity.

### T cell–specific Crebzf deficiency ameliorates diet-induced metabolic disturbance and epididymal adipose tissue inflammation.

**Figure 2:**
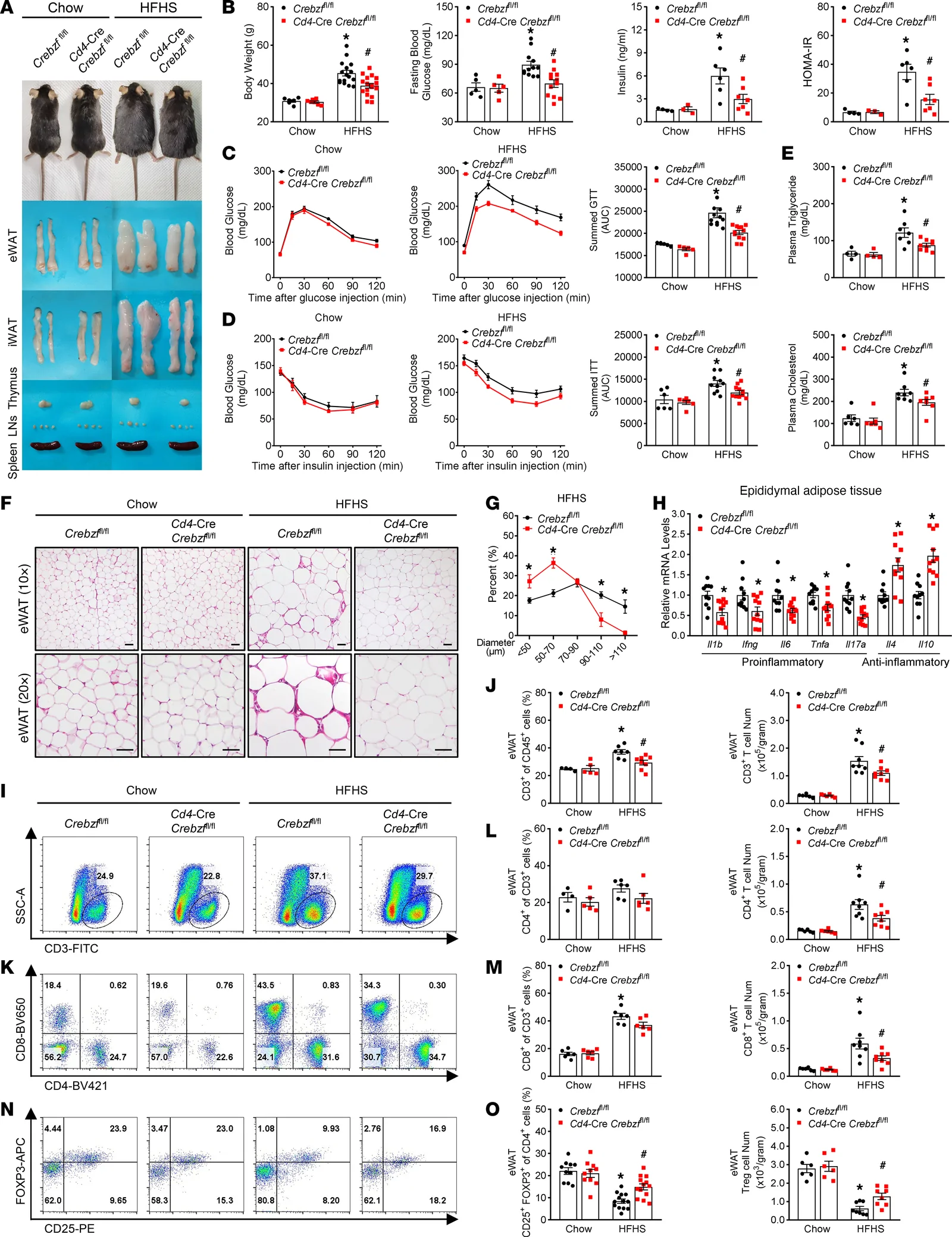
T cell–specific *Crebzf* deficiency protects against HFHS diet–induced metabolic disorders and eWAT inflammation in mice. Male *Cd4*-Cre *Crebzf^fl/fl^* and control littermates *Crebzf^fl/fl^* mice at 8 weeks were fed a chow or HFHS diet for 16 weeks. (**A**) Representative appearance of mice and tissues of *Crebzf^fl/fl^* and *Cd4*-Cre *Crebzf^fl/fl^* mice fed a chow or HFHS diet. (**B**) Body weight, fasting blood glucose levels, plasma insulin levels, and HOMA-IR. (**C**) GTT results and AUC. (**D**) ITT results and AUC. (**E**) Plasma triglyceride and cholesterol levels. (**B**–**E**) Data are presented as the mean ± SEM. Chow-fed group, *n* = 3–7; HFHS-fed group, *n* = 6–17. Two-way ANOVA for multiple comparison. *P < 0.05 versus *Crebzf^fl/fl^* and chow diet; ^#^*P* < 0.05 versus *Crebzf^fl/fl^* and HFHS diet. (**F**) Representative H&E staining of eWAT from mice fed a chow or HFHS diet. Scale bars: 50 μm. (**G**) Quantification of adipocyte diameter of HFHS-fed mice. *n* = 6. (**H**) mRNA levels of proinflammatory and antiinflammatory genes in eWAT of mice fed a HFHS diet. *n* = 10–11. (**G** and **H**) Two-tailed, unpaired Student’s *t* test. *P < 0.05 versus *Crebzf^fl/fl^*. (**I** and **J**) Representative flow cytometric plots (**I**) and quantification of frequencies and numbers (**J**) of CD3^+^ T cells in eWAT from *Crebzf^fl/fl^* and *Cd4*-Cre *Crebzf^fl/fl^* mice fed a chow or HFHS diet. (**K**–**M**) Representative flow cytometric plots (**K**) and quantification of the frequencies and numbers of CD4^+^ (**L**) and CD8^+^ (**M**) T cells in eWAT from mice. (**N** and **O**) Representative flow cytometric plots (**N**) and quantification of frequencies and numbers (**O**) of Tregs in eWAT from mice. Data are presented as the mean ± SEM. Chow group, *n* = 4–10; HFHS group, *n* = 6–13. Two-way ANOVA for multiple comparison. *P < 0.05 versus *Crebzf^fl/fl^* and chow; ^#^*P* < 0.05 versus *Crebzf^fl/fl^* and HFHS.

To assess the T cell–intrinsic function of CREBZF in adipose tissue remodeling during obesity, T cell–specific *Crebzf*-KO mice were generated (Supplemental Figure 2A). As expected, the mRNA and protein levels of CREBZF were markedly reduced in T cells, but not in B cells from *Cd4*-Cre *Crebzf^fl/fl^* mice (Supplemental Figure 2, B and C). *Cd4*-Cre *Crebzf^fl/fl^* mice exhibited no significant difference in metabolic characteristics compared with control littermates under chow diet conditions (Figure 2, A–E, and Supplemental Figure 2, D–G). Strikingly, *Crebzf* deficiency attenuated HFHS diet–induced metabolic disorders, including improved glucose tolerance, reduced hyperglycemia, and insulin resistance (Figure 2, A–D). Furthermore, *Crebzf* KO modestly reduced plasma triglyceride and total cholesterol levels (Figure 2E) and body weight, with decreased fat mass but no changes in lean mass (Supplemental Figure 2, H–L). These results demonstrate that T cell *Crebzf* deficiency alleviated HFHS diet–induced systemic metabolic disturbances, establishing the role of *Crebzf* in linking T cell–intrinsic function to obesity-associated metabolic regulation.

Pathological remodeling and functional alterations of WAT mediated by T cells play crucial roles in the progression of obesity-related metabolic diseases (6, 7, 32). We analyzed the histology, cellularity, and microenvironment of eWAT to assess whether *Crebzf* deficiency in T cells reduces inflammation and improves metabolic parameters. *Cd4*-Cre *Crebzf^fl/fl^* mice exhibited markedly improved obesity-associated pathology in eWAT, including reduced adipocyte size, crown-like structure (CLS) accumulation, and immune cell infiltration (Figure 2, F and G). This histologic improvement correlated with a shift in the expression of inflammatory markers in eWAT, where proinflammatory genes were downregulated and antiinflammatory genes were upregulated (Figure 2H). Flow cytometric analysis showed that T cell *Crebzf* deficiency had little effect on T cell populations in mice fed a chow diet, as demonstrated by similar frequencies and numbers of CD3^+^, CD4^+^, and CD8^+^ T cells (Figure 2, I–M). A dramatic reduction in the frequencies and total number of CD3^+^, CD4^+^, and CD8^+^ T cells was observed in eWAT of *Cd4*-Cre *Crebzf^fl/fl^* mice with HFHS diet (Figure 2, I–M). Notably, eWAT Treg accumulation was not defective in *Cd4*-Cre *Crebzf^fl/fl^* mice fed a chow diet but became impaired under obese conditions. Moreover, *Crebzf* deficiency in T cells specifically restored the HFHS diet–induced loss of Tregs in eWAT, significantly increasing both their percentage and absolute number (Figure 2, N and O). In contrast, the populations of Th1 and Th17 CD4^+^ T cells were unaltered by *Crebzf* deletion (Supplemental Figure 3, A–D), indicating that the attenuated inflammation in eWAT was selectively mediated by Tregs. We further confirmed that *Crebzf* deficiency did not disrupt Treg homeostasis in other tissues (Supplemental Figure 3, E and F). Taken together, these data establish that *Crebzf* deficiency remodels the T cell landscape in eWAT and that the subsequent attenuation of tissue inflammation is specifically dependent on Treg-mediated suppression.

### Treg-intrinsic Crebzf is required for obesity-induced metabolic dysfunction and eWAT inflammation.

To validate whether CREBZF mediates the effects on inflammation and metabolism directly through Tregs, Treg-specific deficient mice (*Foxp3*-Cre *Crebzf^fl/fl^*) and control mice (*Foxp3*-Cre) were generated. *Crebzf*-sufficient (*Foxp3*-Cre) and *Crebzf*-deficient (*Foxp3*-Cre *Crebzf^fl/fl^*) mice express yellow fluorescent protein (YFP), which serves as a reporter for *Foxp3* expression in Tregs (33). We verified that CREBZF expression was specifically abolished in Tregs but not in CD4^+^ conventional T (Tconv) cells (Supplemental Figure 4, A–C).

**Figure 3:**
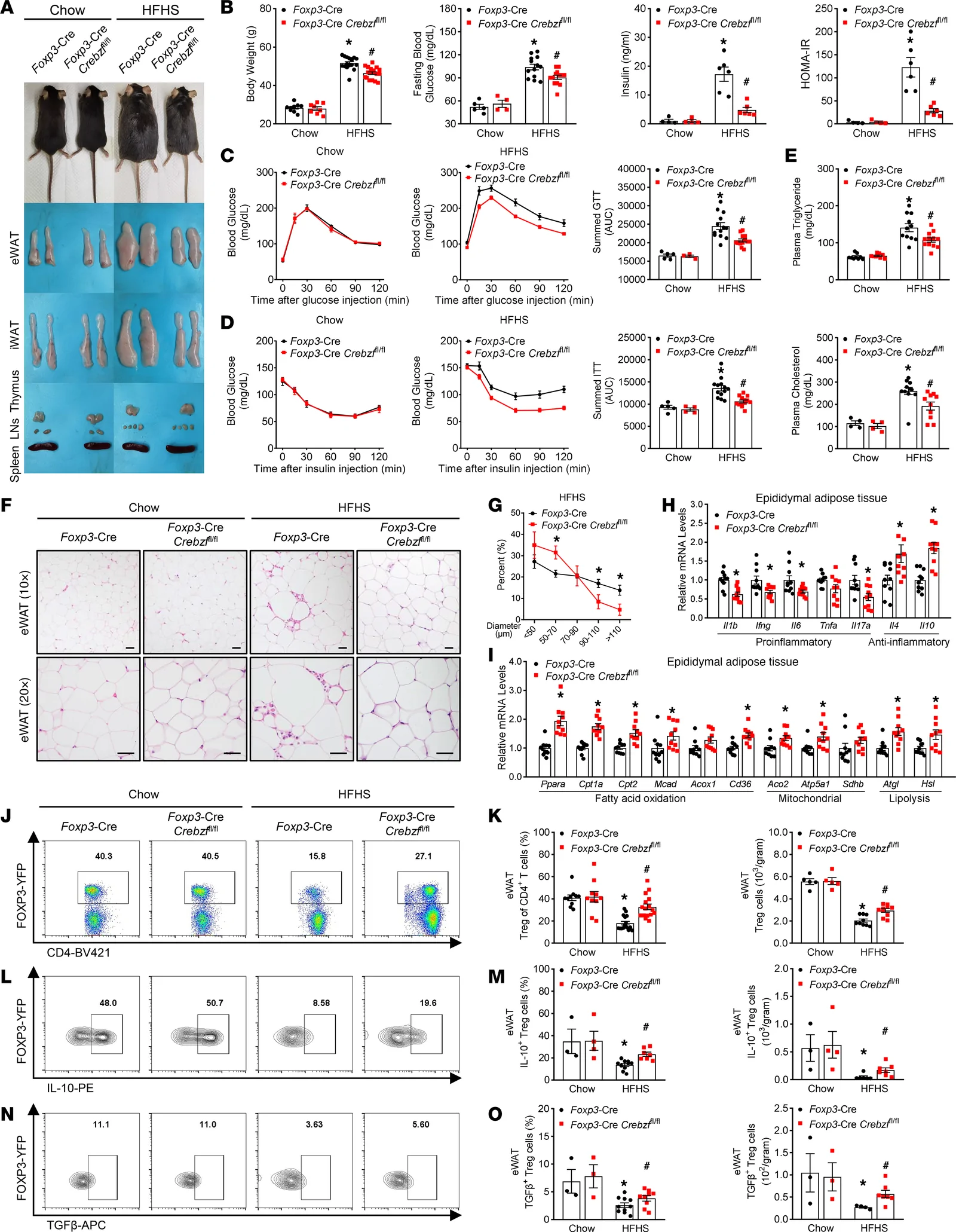
CREBZF in Tregs links eWAT inflammation to systemic metabolic dysfunction during obesity. Male *Foxp3*-Cre *Crebzf^fl/fl^* and control *Foxp3*-Cre mice at 8 weeks were fed a chow or HFHS diet for 16 weeks. (**A**) Representative appearance of mice and tissues of *Foxp3*-Cre and *Foxp3*-Cre *Crebzf^fl/fl^* mice fed a chow or HFHS diet. (**B**) Body weight, fasting blood glucose levels, plasma insulin levels, and HOMA-IR. (**C**) GTT results and AUC. (**D**) ITT results and AUC. (**E**) Plasma triglyceride and cholesterol levels. (**B**–**E**) Data are presented as the mean ± SEM. Chow group, *n* = 4–9; HFHS group, *n* = 6–13. Two-way ANOVA for multiple comparison. *P < 0.05 versus *Foxp3*-Cre and chow; ^#^*P* < 0.05 versus *Foxp3*-Cre and HFHS. (**F**) Representative H&E staining of eWAT from *Foxp3*-Cre and *Foxp3*-Cre *Crebzf^fl/fl^* mice fed a chow or HFHS diet. Scale bars: 50 μm. (**G**) Quantification of diameters of adipocytes from mice. *n* = 4–5. (**H** and **I**) Relative mRNA levels of inflammatory (**H**), lipid metabolism–, and mitochondria-related (**I**) genes in eWAT of mice fed a HFHS diet. (**G**–**I**) Data are presented as the mean ± SEM. *n* = 4–11. Two-tailed, unpaired Student’s *t* test. *P < 0.05 versus *Foxp3*-Cre. (**J** and **K**) Representative flow cytometric plots (**J**) and quantification of frequencies and numbers (**K**) of Tregs in eWAT of mice. (**L** and **M**) Representative flow cytometric plots (**L**) and quantification of frequencies and numbers (**M**) of IL-10^+^ Tregs in eWAT of mice. (**N** and **O**) Representative flow cytometric plots (**N**) and quantification of frequencies and numbers (**O**) of TGF-β^+^ Tregs in eWAT of mice. Data are presented as the mean ± SEM. Chow group, *n* = 3–10; HFHS group, *n* = 4–16. Two-way ANOVA for multiple comparison. *P < 0.05 versus *Foxp3*-Cre and chow; ^#^*P* < 0.05 versus *Foxp3*-Cre and HFHS.

Consistent with the findings in *Cd4*-Cre *Crebzf^fl/fl^* mice (Figure 2), Treg-intrinsic ablation of *Crebzf* was sufficient to improve systemic metabolic parameters under HFHS diet conditions (Figure 3, A–E, and Supplemental Figure 4, D–L). Furthermore, *Foxp3*-Cre *Crebzf^fl/fl^* mice displayed ameliorated eWAT pathology, characterized by reduced adipocyte hypertrophy and diminished inflammatory cell accumulation (Figure 3, F and G). Consistent with this improvement in tissue pathology, the expression of proinflammatory genes was reduced and antiinflammatory genes were elevated in eWAT of HFHS diet–fed *Foxp3*-Cre *Crebzf^fl/fl^* mice (Figure 3H). Additionally, eWAT from *Foxp3*-Cre *Crebzf^fl/fl^* mice showed an upregulation of gene programs for fatty acid oxidation, mitochondrial biogenesis, and lipolysis (Figure 3I). Moreover, the frequencies of CD3^+^, CD4^+^, and CD8^+^ T cells and total macrophages showed reduced accumulation in eWAT from *Foxp3*-Cre *Crebzf^fl/fl^* mice fed a HFHS diet (Supplemental Figure 5, A–G). Interestingly, under HFHS diet conditions, Treg-specific *Crebzf* deficiency significantly reduced the proportion of the TREM2^+^ lipid–associated macrophage (LAM) subset in eWAT, which has emerged as a key player in obesity and in diseases involving lipid stress and inflammation (Supplemental Figure 5H). These results collectively demonstrate a broad amelioration of both inflammatory and metabolic dysfunction in eWAT upon *Crebzf* deficiency.

Given that adipose tissue Tregs suppress local inflammation and improve metabolic homeostasis via the secretion of IL-10 and TGF-β (34), we analyzed Treg abundance and function in our model. Under a chow diet, *Foxp3*-Cre *Crebzf^fl/fl^* and *Foxp3*-Cre mice showed no difference in eWAT Treg abundance. In contrast, upon HFHS diet feeding, *Foxp3*-Cre *Crebzf^fl/fl^* mice showed a significant increase in both the frequency and number of Tregs within eWAT (Figure 3, J and K). This expansion included a greater proportion of cells producing IL-10 and TGF-β (Figure 3, L–O), indicating enhanced stability and suppressive function of *Crebzf*-deficient Tregs in obesity. Moreover, *Crebzf* deficiency did not disrupt the balance in other tissues of *Foxp3*-Cre *Crebzf^fl/fl^* mice fed a chow or HFHS diet (Supplemental Figure 5, I and J). Taken together, these results indicate that *Crebzf* deletion specifically enhances eWAT Treg fitness by bolstering the suppressive capacity and stability of these cells.

### PA impairs Treg stability by upregulating CREBZF.

**Figure 4:**
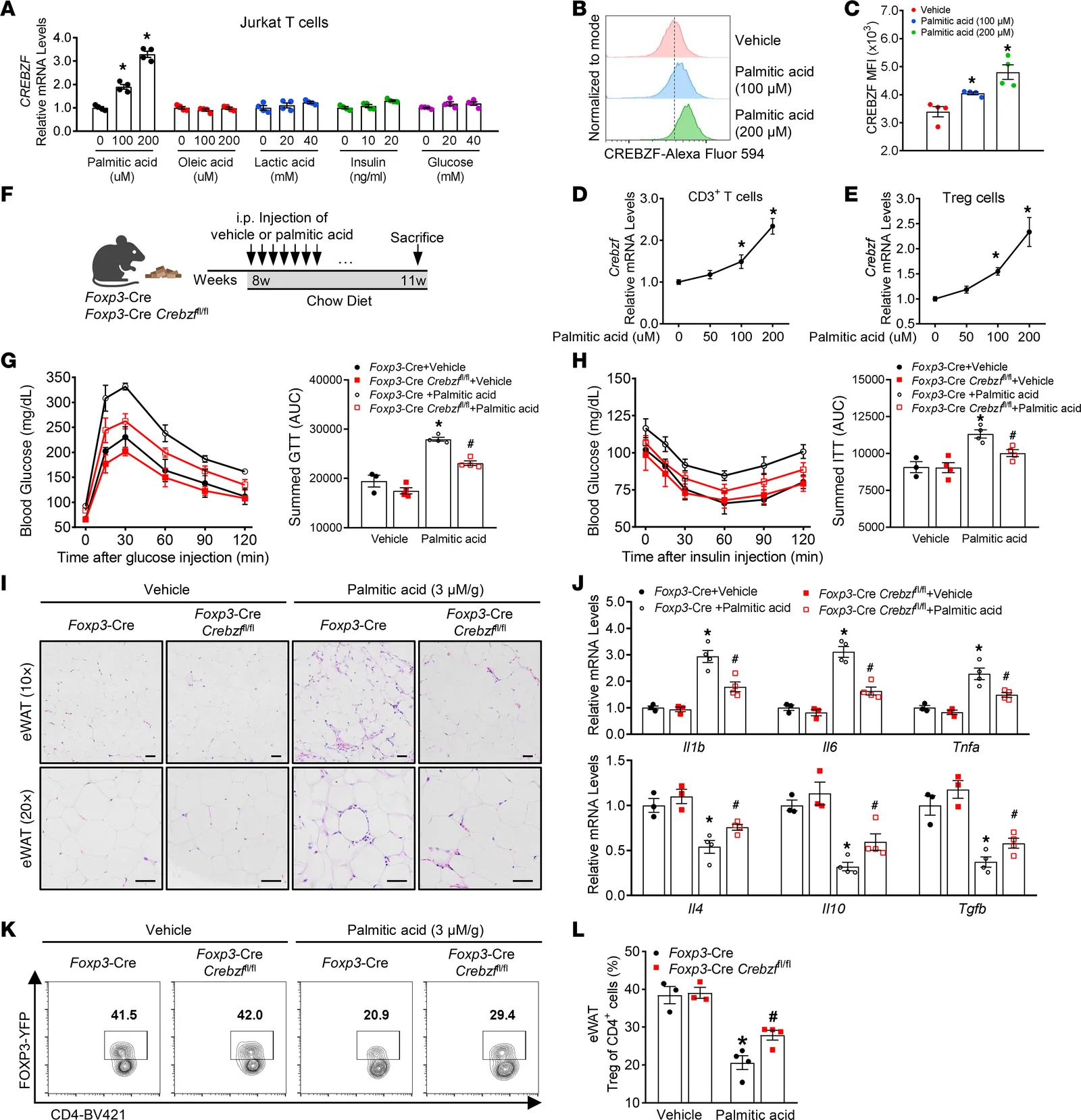
PA impairs Treg-mediated immunosuppression via CREBZF upregulation. (**A**–**D**) PA upregulates *CREBZF* expression at the mRNA and protein levels. (**A**) Quantitative PCR (qPCR) analysis of *CREBZF* expression in Jurkat T cells following treatment with different concentrations of PA, oleic acid, lactic acid, insulin, glucose, or vehicle for 24 hours. *n* = 4. (**B** and **C**) Representative histograms (**B**) and MFI (**C**) of *CREBZF* in Jurkat T cells treated with the indicated concentrations of PA or vehicle for 24 hours. *n* = 4. (**D** and **E**) CD3^+^ T cells (**D**) and Tregs (**E**) were isolated from spleens and lymph nodes of mice and treated with PA or vehicle as indicated for 24 hours. Relative *Crebzf* mRNA levels are shown. *n* = 6. (**A**–**E**) Data are presented as the mean ± SEM. One-way ANOVA for multiple comparison. *P < 0.05 versus vehicle. (**F**–**H**) Male *Foxp3*-Cre *Crebzf^fl/fl^* and control *Foxp3*-Cre mice fed a chow diet were administered daily i.p. injections of PA (ethyl palmitate, 3 μM/g) or vehicle for 3 weeks. *n* = 3–4. (**F**) Schematic diagram of the animal experiment. (**G**) GTT results and AUC. (**H**) ITT results and AUC. (**I**) Representative H&E staining of eWAT from *Foxp3*-Cre and *Foxp3*-Cre *Crebzf^fl/fl^* mice injected with PA or vehicle. Scale bars: 50 μm. (**J**) Relative mRNA levels of inflammatory genes in eWAT. (**K** and **L**) Representative flow cytometric plots (**K**) and quantification of frequencies (**L**) of Tregs in eWAT from *Foxp3*-Cre and *Foxp3*-Cre *Crebzf^fl/fl^* mice injected with PA or vehicle. (**G**–**L**) Data are presented as the mean ± SEM. *n* = 3–4. Two-way ANOVA for multiple comparison. *P < 0.05 versus *Foxp3*-Cre and vehicle; ^#^*P* < 0.05 versus *Foxp3*-Cre and PA.

VAT is enriched with a diverse array of metabolites, hormones, and inflammatory signals (35, 36). Consequently, resident immune cells undergo distinct adaptations in response to the shifting physiological and pathological landscape of obesity (37). To investigate whether obesity-associated pathological adaptations of CREBZF in T cell subsets within eWAT are driven by the nutrient overload, we screened metabolites and hormones enriched in obese VAT for their ability to regulate CREBZF. Treatment of Jurkat T cells with PA, but not oleic acid, lactic acid, insulin, or high glucose, induced a robust and dose-dependent upregulation of CREBZF at both mRNA and protein levels (Figure 4, A–C, and Supplemental Figure 6A). Moreover, this induction by PA was further confirmed in primary mouse CD3^+^ T cells and Tregs (Figure 4, D and E). Consistent with our observation that PA exerts an inductive effect, analysis of the Gene Expression Omnibus (GEO) public database (GSE174706) revealed that long-term high-fat diet feeding significantly induced *Crebzf* expression at the transcriptional level (Supplemental Figure 6B). In contrast, T cell activation did not alter *CREBZF* expression (Supplemental Figure 6, C–H).

To further evaluate the effects of PA in vivo, male *Foxp3*-Cre *Crebzf^fl/fl^* and control *Foxp3*-Cre mice on a chow diet received daily i.p. injections of PA (3 μM/g) or vehicle for 3 weeks (Figure 4F). PA-treated mice displayed aggravated metabolic deficits, as evidenced by impaired glucose tolerance and insulin sensitivity (Figure 4, G and H). Three weeks of PA injection moderately increased body weight in mice fed a chow diet, and *Crebzf* deficiency slightly attenuated this weight gain (Supplemental Figure 6I). Concomitantly, H&E-stained histological sections of eWAT revealed aggravated pathology characterized by enhanced immune cell infiltration, increased CLSs, and tissue disruption (Figure 4I). Accordingly, the eWAT inflammatory profile was shifted, with upregulated proinflammatory and downregulated antiinflammatory gene expression (Figure 4J). Importantly, Treg-specific deletion of *Crebzf* significantly abrogated the PA-induced glycolipid metabolic disorder, pathological deterioration, and inflammation (Figure 4, G–J).

PA generally induces polarization of proinflammatory T cell subsets and is considered to decrease the proportion of Tregs and the stability of FOXP3 (23, 24, 38). As shown in Figure 4, K and L, PA treatment significantly reduced the frequencies of Tregs in eWAT, a loss that was reversed by *Crebzf* KO. Taken together, these results demonstrate that PA-dependent overactivation of CREBZF was sufficient to impair Treg stability, thereby contributing to metabolic disturbance and inflammation in eWAT during obesity progression.

### Identification of 2 distinct Treg subsets in epididymal adipose tissue.

The VAT Treg compartment exhibits considerable heterogeneity and contributes to the maintenance of beige fat biogenesis, insulin sensitivity, and systemic homeostasis (17–19). Therefore, the observed metabolic and inflammatory improvements upon *Crebzf* deletion could be attributed either to alterations in a specific VAT Treg subset or to broader reprogramming across the entire Treg compartment. To this end, we performed single-cell RNA-seq (scRNA-seq) using the 10X Genomics platform on Tregs isolated from eWAT of HFHS diet–fed mice. An unbiased integrative analysis across 2 genotypes after regressing out batch effects and other confounding factors using the Seurat R package yielded 17,702 eWAT Tregs (8,091 from control mice and 9,611 from *Foxp3*-Cre *Crebzf^fl/fl^* mice).

**Figure 5:**
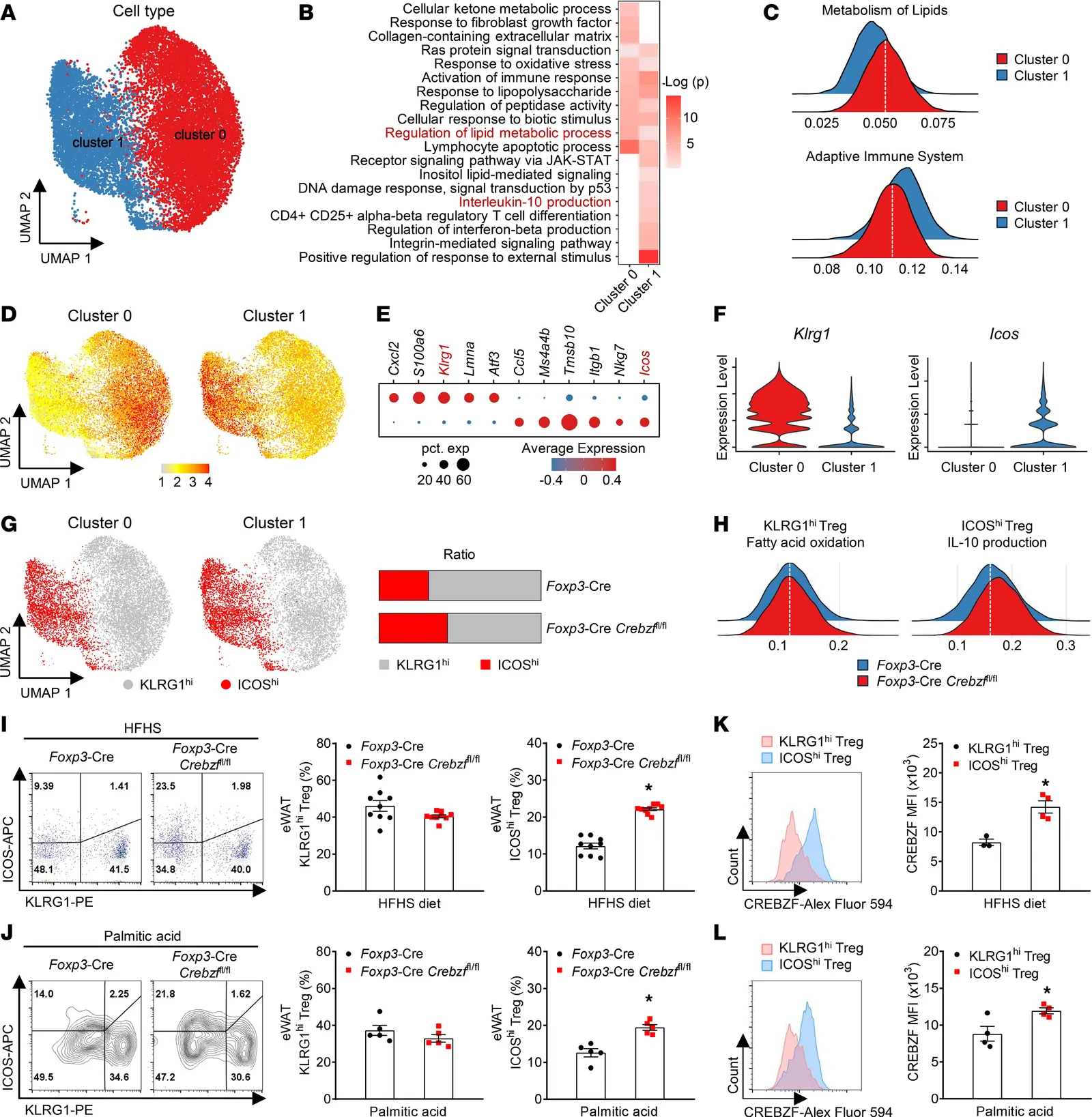
scRNA-seq identifies distinct KLRG1^hi^ and ICOS^hi^ Treg subsets in epididymal adipose tissue. (**A**) UMAP representation of 17,702 eWAT Tregs color-coded by subset. (**B**) GO enrichment analysis of the DEGs between Treg clusters 0 and 1. (**C**) Ridge plots depicting the functional scores from the indicated Treg subtypes using AUCell. (**D**) UMAP visualization of signature genes of cluster 0 and cluster 1 Tregs from scRNA-seq. (**E**) Dot plot of top marker genes for the 2 Treg subsets. (**F**) Violin plot aligning *Klrg1* and *Icos* expression at the transcriptional level for Treg subsets. (**G**) UMAP representations and cluster composition of *Crebzf*-sufficient (*n* = 2) and *Crebzf*-deficient (*n* = 2) Tregs. (**H**) Ridge plots of fatty acid oxidation in the KLRG1^hi^ Treg cluster and IL-10 production in the ICOS^hi^ cluster, color-coded by sample condition. (**I**) Representative flow cytometric plots and frequencies of KLRG1^hi^ and ICOS^hi^ Tregs in eWAT from *Foxp3*-Cre and *Foxp3*-Cre *Crebzf^fl/fl^* mice fed a HFHS diet. *n* = 9. (**J**) Representative flow cytometric plots and frequencies of KLRG1^hi^ and ICOS^hi^ Tregs in eWAT from *Foxp3*-Cre and *Foxp3*-Cre *Crebzf^fl/fl^* mice treated with PA. *n* = 5. (**I** and **J**) Data are presented as the mean ± SEM. Two-tailed, unpaired Student’s *t* test. *P < 0.05 versus *Foxp3*-Cre. (**K**) Representative histograms and MFI of CREBZF in KLRG1^hi^ (pink) and ICOS^hi^ (blue) Tregs from *Foxp3*-Cre mice fed a HFHS diet. *n* = 3–4. (**L**) Representative histograms and MFI of CREBZF in KLRG1^hi^ (pink) and ICOS^hi^ (blue) Tregs from *Foxp3*-Cre mice treated with PA. *n* = 4. (**K** and **L**) Data are presented as the mean ± SEM. Two-tailed, unpaired Student’s *t* test. *P < 0.05 versus KLRG1^hi^.

Analysis based on differentially expressed genes (DEGs) reliably divided eWAT Tregs into subsets with metabolic and immune signatures, confirming the distinct features of the 2 subsets on a uniform manifold approximation and projection (UMAP) representation (Figure 5A and Supplemental Figure 7A). To further define discrete subsets of Tregs, we performed Gene Ontology (GO) pathway analysis using DEGs. Cluster 0 highlighted pathways indicating intracellular metabolism, whereas cluster 1 was enriched for pathways involved in immune response (Figure 5B). Moreover, ridge plots depicting profile scores for lipid metabolism and adaptive immune response in each cluster demonstrated that these 2 Treg subsets exhibited different functional preferences (Figure 5C). These subsets were annotated according to the most salient identified cell markers (Figure 5, D and E). Tregs from cluster 0 exhibited high expression of killer cell lectin–like receptor G1 (KLRG1), a transmembrane glycoprotein that is associated with an activated and memory phenotype, and were accordingly termed KLRG1^hi^ Tregs. In addition, cluster 1 Tregs showed prominent expression of inducible T cell costimulator (ICOS). These were therefore termed ICOS^hi^ Tregs (Figure 5F and Supplemental Figure 7B).

We next examined the alterations in the proportions of KLRG1^hi^ and ICOS^hi^ Tregs under chow and HFHS diet conditions. As shown in Supplemental Figure 7C, HFHS diet feeding significantly increased the frequencies of ICOS^hi^ Tregs and decreased those of KLRG1^hi^ Tregs. Next, to determine the contribution of CREBZF to Treg identity and function, a side-by-side comparison of the scRNA-seq data from *Foxp3*-Cre *Crebzf^fl/fl^* and *Foxp3*-Cre mice was conducted. Compared with control mice, loss of *Crebzf* increased the frequency of ICOS^hi^ Tregs, whereas the proportion of KLRG1^hi^ Tregs was slightly decreased (Figure 5G). Furthermore, pathway analysis revealed that *Crebzf* deficiency specifically enhanced the IL-10 production pathway in ICOS^hi^ Tregs, whereas it did not significantly alter the fatty acid oxidation pathway in KLRG1^hi^ Tregs (Figure 5H). Consistently, GO analysis of upregulated genes indicated that *Crebzf*-deficient ICOS^hi^ Tregs possessed a distinctly elevated immunosuppressive transcriptional signature and enhanced functional capacity (Supplemental Figure 7, D and E). These findings suggest that the increase in ICOS^hi^ Tregs upon *Crebzf* KO may represent a protective mechanism.

To further confirm the scRNA-seq findings, we performed flow cytometric analysis using the cluster-defining markers KLRG1 and ICOS. We observed that *Crebzf* deficiency significantly increased the frequency and number of ICOS^hi^ Tregs under both HFHS diet and palmitate treatment conditions, while it slightly elevated the number of KLRG1^hi^ Tregs in HFHS diet–fed mice (Figure 5, I and J, and Supplemental Figure 7F). Importantly, flow cytometric and mRNA abundance analysis showed that *Crebzf* was specifically expressed in ICOS^hi^ Tregs, suggesting that CREBZF is a key mediator preventing ICOS^hi^ Treg accumulation in VAT during the progression of obesity (Figure 5, K and L). Notably, *Crebzf* deficiency did not significantly alter the frequency or number of the ICOS^hi^ Treg subset in inguinal WAT (iWAT) or spleen (Supplemental Figure 7, G–L), suggesting that the function of CREBZF is eWAT specific. Interestingly, a dietary switch from a high-fat diet to chow and caloric restriction–induced (CR-induced) weight loss also increased the proportion of Tregs in mouse eWAT, particularly ICOS^hi^ Tregs (Supplemental Figure 8), which is consistent with the changes in Treg subsets observed upon *Crebzf* KO, further supporting our finding that ICOS^hi^ Tregs played a protective role under obese pathological conditions. Taken together, our findings reveal 2 distinct eWAT Treg subsets with unique transcriptional and functional identities. Furthermore, we demonstrate that *Crebzf* specifically sustained the fitness of the ICOS^hi^ Treg subset in the obese eWAT microenvironment.

### Adoptive transfer of Crebzf-deficient ICOS^hi^ Tregs attenuates the severity of HFHS diet–induced inflammation and metabolic disorders.

**Figure 6:**
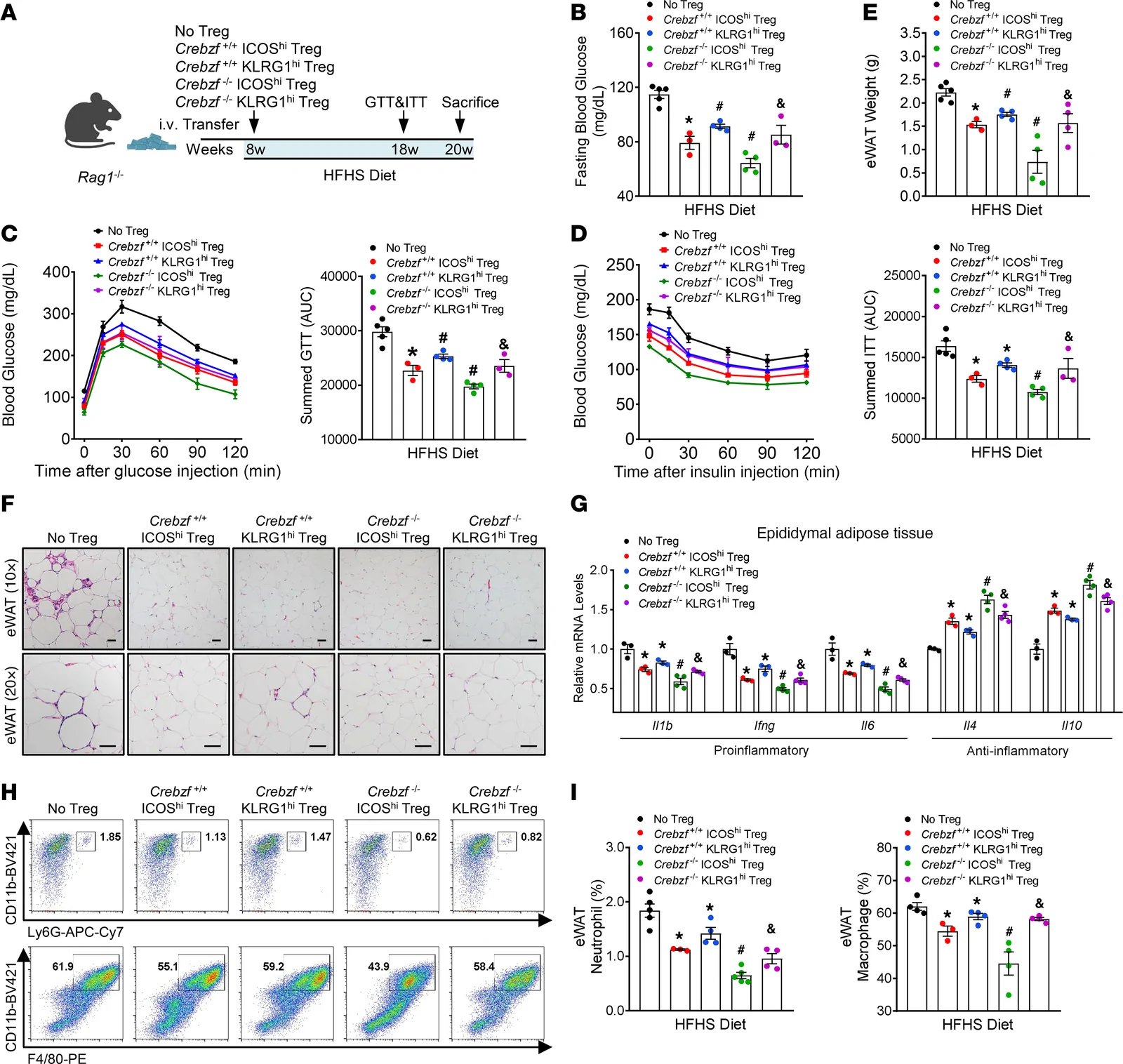
*Crebzf*-deficient ICOS^hi^ Tregs suppress eWAT inflammation. In an adoptive transfer model, *Crebzf*-sufficient or -deficient ICOS^hi^ or KLRG1^hi^ Tregs were transferred into *Rag1^–/–^* recipients, followed by subsequent HFHS diet feeding for 12 weeks. *n* = 3–5. (**A**) Schematic diagram of the animal experiment. (**B**) Fasting blood glucose levels. (**C**) GTT results and AUC. (**D**) ITT results and AUC. (**E**) eWAT weights. (**F**) Representative H&E staining of eWAT from *Rag1^–/–^* mice transferred with Tregs followed by HFHS diet feeding. Scale bars: 50 μm. (**G**) Relative mRNA levels of inflammatory genes in eWAT. (**H** and **I**) Representative flow cytometric plots (**H**) and frequencies (**I**) of CD11c^+^Ly6G^+^ neutrophils and F4/80^+^ CD11b^+^ macrophages in eWAT from *Rag1^–/–^* mice transferred with Tregs followed by HFHS diet feeding. Data are presented as the mean ± SEM. *n* = 3–5. One-way ANOVA for multiple comparison. *P < 0.05 versus no Tregs; ^#^*P* < 0.05 versus *Crebzf* ^+/+^ ICOS^hi^; ^&^*P* < 0.05 versus *Crebzf* *^–/–^* ICOS^hi^.

Given the role of CREBZF in regulating ICOS^hi^ Treg immunosuppression (Figure 5, K and L), we sought to define the precise function and mechanism of CREBZF in Treg subset specialization under metabolic stress. Splenic ICOS^hi^ and KLRG1^hi^ Tregs, either *Crebzf* sufficient (*Crebzf^+/+^*) or *Crebzf* deficient (*Crebzf* *^–/–^*), were sorted and transferred into *Rag1^–/–^* recipients, which were then fed a HFHS diet for 12 weeks (Figure 6A). Metabolic analysis revealed that mice receiving no Tregs developed severe metabolic dysfunction. In contrast, varying degrees of improved glycemic control and insulin sensitivity were observed in mice that received either ICOS^hi^ or KLRG1^hi^ Tregs. Importantly, *Crebzf* deletion further enhanced this metabolic improvement function specifically in the ICOS^hi^ Treg subset, whereas KLRG1^hi^ Tregs showed little change (Figure 6, B–D). Histological analysis revealed reduced eWAT weights and amelioration of obesity-associated pathology in mice receiving *Crebzf* *^–/–^* ICOS^hi^ Tregs, compared with other groups (Figure 6, E and F). Gene expression analysis of eWAT indicated that the transfer of *Crebzf^–/–^* ICOS^hi^ Tregs was associated with reduced expression of proinflammatory cytokine genes and increased levels of antiinflammatory markers (Figure 6G). The infiltration of macrophages and neutrophils is a hallmark of obese eWAT (4, 39). Flow cytometric analysis revealed that adoptive transfer of *Crebzf* *^–/–^* ICOS^hi^ Tregs into *Rag1^–/–^* mice significantly reduced the proportions of both neutrophils and macrophages in eWAT, indicating a potent attenuation of obesity-associated adipose tissue inflammation (Figure 6, H and I). These findings establish the idea that *Crebzf* deficiency enhances the ability of ICOS^hi^ Tregs to restrain systemic metabolic dysregulation and eWAT inflammation induced by a HFHS diet.

We next investigated the mechanism by which CREBZF regulates the stability and suppressive function of ICOS^hi^ Tregs. The assay for transposase accessible chromatin with high-throughput sequencing (ATAC-seq) was performed to assess its role in modulating chromatin accessibility on eWAT Tregs. *Crebzf*-deficient Tregs showed increased chromatin accessibility compared with *Crebzf*-sufficient cells (Supplemental Figure 9A). Specifically, following *Crebzf* deletion, chromatin accessibility increased more markedly at signature loci of ICOS^hi^ Tregs (such as *Icos* and *Tigit*) compared with those of KLRG1^hi^ Tregs (including *Klrg1* and *Pparg*) (Supplemental Figure 9B). The top-8 ranked motifs correspond to binding of the activator protein 1 (AP-1) family members generated based on Hypergeometric Optimization of Motif Enrichment (HOMER) (Supplemental Figure 9C).

*Crebzf* is established as a transcriptional coregulator that does not bind DNA directly (30). Cofactors of CREBZF were screened using our scRNA-seq dataset, and a series of transcription factors exhibited differential activity specifically in ICOS^hi^ Tregs (Supplemental Figure 9D). Co-immunoprecipitation analysis demonstrated that CREBZF directly interacted with c-JUN, the major component of the AP-1 family (40), and we detected the intracellular colocalization between c-JUN and CREBZF in Tregs (Supplemental Figure 9, E–G). AP-1 is a dimeric transcription factor composed of members from the JUN, FOS, ATF, and MAF protein families. Its activity is regulated through dimer composition, transcriptional control, posttranslational modifications, and interactions with other proteins (41). Overexpression of CREBZF attenuated the interaction between c-FOS and c-JUN, which suggested that CREBZF suppressed the AP-1 activity (Supplemental Figure 9H). In response to TCR and costimulatory signaling, the *Foxp3* promoter is bound and activated by transcription factors such as nuclear factors of activated T cells (NFAT) and AP‑1 (10, 42). Given the observation that *Crebzf* deficiency significantly increased the expression of *Foxp3* in ICOS^hi^ Tregs (Supplemental Figure 7D) and enhanced AP-1 family factor activity (Supplemental Figure 9C), we propose that CREBZF may regulate *Foxp3* expression in ICOS^hi^ Tregs, and consequently their stability, by restricting AP-1 transcription factor activity. Next, we confirmed that CREBZF inhibited AP-1–driven *Foxp3* luciferase activity in a dose-dependent manner (Supplemental Figure 9I). We next elucidated the causal relationship between c-JUN- and CREBZF-mediated inhibition of *Foxp3* and the suppressive function of ICOS^hi^ Tregs. As expected, the suppressive effects of CREBZF on the transcription levels of *Foxp3*, *Il10*, and *Tgfb* were reduced by c-JUN siRNA in ICOS^hi^ Tregs (Supplemental Figure 9, J and K). Taken together, these results demonstrate that CREBZF bound to c-JUN to reduce interaction between c-JUN and c-FOS (two major components of the AP-1 family), and consequently inhibited the transcriptional activity of *Foxp3*, thereby impairing the stability and suppressive function of ICOS^hi^ Tregs.

### Negative correlation of FOXP3 and suppressive gene expression with elevated CREBZF in VAT.

**Figure 7:**
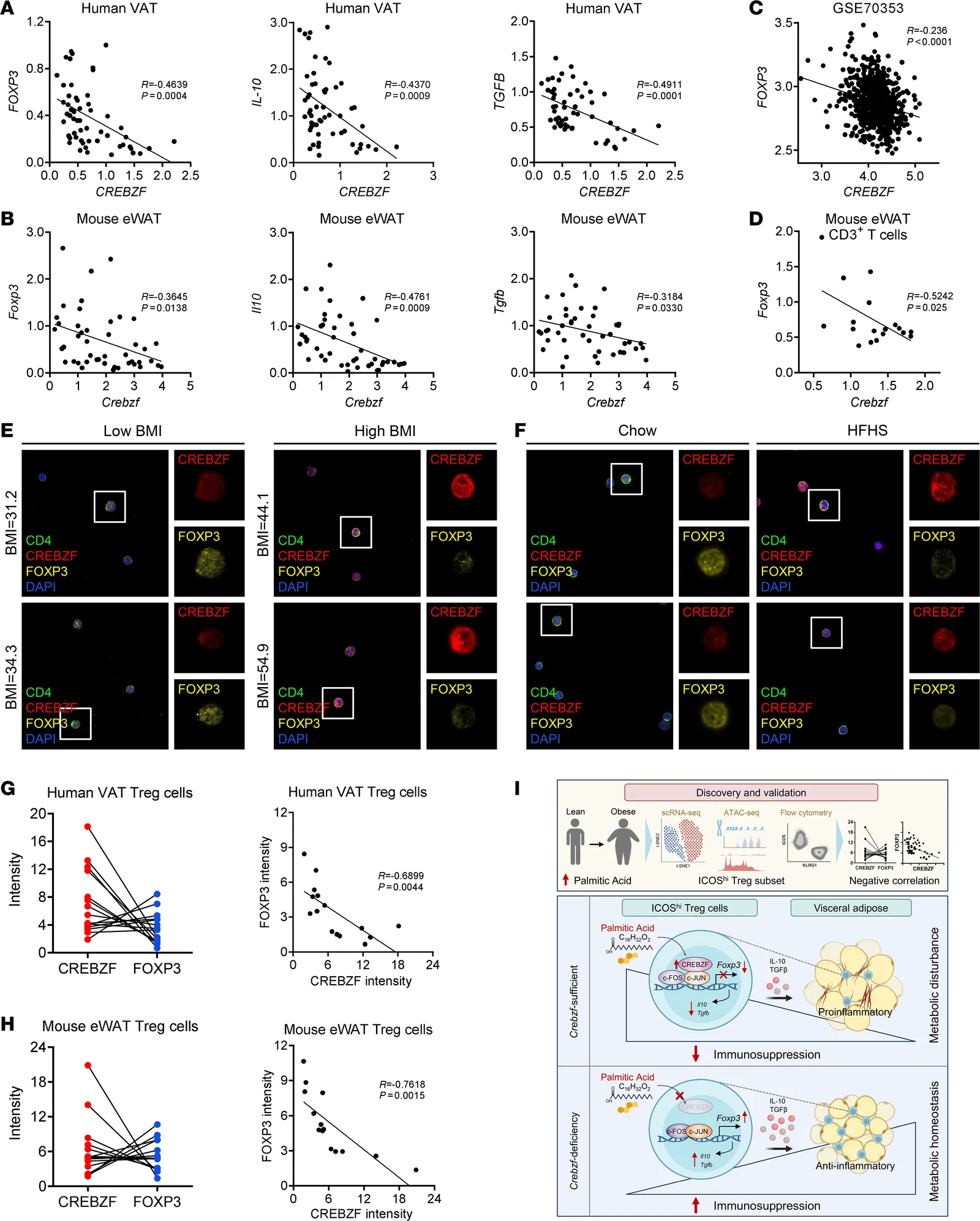
CREBZF inversely correlates with suppressive gene expression in Tregs from human VAT and mouse eWAT. (**A**–**D**) Pearson correlation analyses of mRNA levels in VAT. (**A**) Correlation of *CREBZF* with *FOXP3*, *IL10*, and *TGFB* expression in VAT from individuals with obesity. *n* = 55. (**B**) Correlation of *Crebzf* with *Foxp3*, *Il10*, and *Tgfb* in mouse eWAT. *n* = 45. (**C**) Correlation of *CREBZF* with *FOXP3* in the GEO public dataset GSE70353. (**D**) Correlation of *Crebzf* with *Foxp3* in CD3^+^ T cells sorted from mouse eWAT. *n* = 18. (**E**) Representative immunofluorescence images of Tregs sorted from VAT from the indicated individuals. Original magnification: x 630. Scale bars: 25 µm (square side length). (**F**) Representative immunofluorescence images of Tregs sorted from mouse eWAT from the indicated mice. Original magnification: x 630. Scale bars: 25 µm (square side length). (**G**) Quantification and correlation analysis of CREBZF and FOXP3 fluorescence intensity in Tregs sorted from human VAT. *n* = 15. (**H**) Quantification and correlation analysis of CREBZF and FOXP3 fluorescence intensity in Tregs sorted from eWAT of mice. *n* = 14. (**I**) Proposed model illustrating the mechanism by which PA mediates immunosuppression of ICOS^hi^ Tregs, thereby regulating VAT remodeling and systemic metabolic homeostasis. During the progression of obesity, PA-induced hyperactivation of CREBZF reduces the frequency and suppressive function of the ICOS^hi^ Treg subset. This occurs through CREBZF binding to c-JUN, which inhibits *Foxp3* transcriptional activity. Therefore, targeting the CREBZF/c-JUN/FOXP3 axis may represent a therapeutic strategy for obesity-related metabolic diseases.

Next, we examined the inhibitory effect of CREBZF on FOXP3 expression and the suppressive function of Tregs in VAT. We detected mRNA levels of *CREBZF* and Treg-suppressive genes (*FOXP3*, *IL10*, and *TGFB*) in VAT from individuals with obesity and in eWAT from mice. We observed a strong negative correlation between *CREBZF* and *FOXP3* and inhibitory cytokine genes in both human VAT and mouse eWAT (Figure 7, A and B). This negative relationship between *Crebzf* and *Foxp3* at the transcript level was also confirmed in a human VAT RNA-seq dataset (GSE70353) and in CD3^+^ T cells sorted from mouse eWAT (Figure 7, C and D).

To further evaluate this observation, we isolated Tregs from human VAT and mouse eWAT and performed flow cytometry and immunofluorescence staining. We found that CREBZF expression was increased in Tregs from the VAT of individuals with a high BMI and in eWAT of HFHS diet–fed mice (Figure 7, E and F). Furthermore, CREBZF expression levels in VAT showed a significant inverse correlation with FOXP3 protein levels (Figure 7, G and H, and Supplemental Figure 10, A and B). Importantly, relative mRNA levels of *CREBZF* and *FOXP3*, *IL10*, and *TGFB* in human SAT and mouse iWAT showed no significant correlation (Supplemental Figure 10, C and D), suggesting a VAT-specific regulatory role of CREBZF. These clinical data further indicate that elevated CREBZF may deteriorate FOXP3 stability and the suppressive function of visceral adipose Tregs during obesity.

## Discussion

Recent studies have highlighted the pivotal role of VAT Tregs in suppressing chronic inflammation and maintaining metabolic homeostasis, thereby extending their known functions beyond classical immune suppression (6, 16, 19, 20, 43). Here, we uncovered the mechanisms by which metabolite signals regulate the heterogeneity and function of VAT Tregs during nutrient-overload and pathological conditions. We identified a specific subset of Tregs characterized by high expression of ICOS (ICOS^hi^ Tregs) that are crucial for restraining inflammation and systemic metabolic dysfunction during obesity. The abundance and suppressive function of this subset were modulated by PA, with the core transcription factor CREBZF acting as a key regulator (Figure 7I).

### PA disrupts VAT Treg homeostasis under obesogenic conditions.

Nutrients and metabolites profoundly shape the immunological and metabolic homeostasis of Tregs in distinct physiological and pathological contexts (23, 24). Succinate acts as a pathogenic factor to trigger a succinylation-to-ubiquitination switch of FOXP3, thereby impairing the immunosuppressive function of Tregs in inflammatory bowel disease (IBD) (44). Similarly, the accumulation of long-chain free fatty acids in the skin exacerbates lipotoxicity and mitochondrial dysfunction in PPARγ^+^ Tregs, impairing their ability to restrain IL-17A^+^ γδ T cells and thereby driving inflammatory progression in psoriasis (38). Our data indicated that PA recapitulated HFHS diet–induced loss of ICOS^hi^ Treg subset stability and exacerbated VAT inflammation and systemic metabolic dysfunction in mice, indicating that PA reshaped Treg homeostasis under obesity pathology. Mechanistically, PA specifically induced CREBZF expression, whereas Treg-specific *Crebzf* deficiency effectively restored Treg stability and blocked this pathogenic cascade. Thus, CREBZF mediated PA-induced impairment of VAT Treg function, linking dietary fatty acids to adipose inflammation and metabolic disease.

Our study establishes CREBZF-mediated PA sensing as a critical pathway that specifically impairs the immunosuppressive function of VAT Tregs under obesogenic conditions, but not that of Tregs in other tissues. First, Treg-specific *Crebzf* deletion selectively increased Treg abundance in VAT without altering Treg homeostasis in other immune or metabolic organs. Second, *Crebzf* deficiency did not cause global immune disruption, as evidenced by normal spleen and lymph node architecture and cellularity. Moreover, the expansion in VAT was attributable to a specific increase in the frequency and number of ICOS^hi^ Tregs.

Tissue-dependent features of VAT may explain this distinctive reliance on PA for Treg regulation. VAT and SAT are functionally divergent (45), differing not only in anatomy but also in their metabolic-inflammatory landscape (46–48). The proinflammatory, saturated fatty acid–rich milieu characteristic of VAT may critically shape the adaptive capacity of Tregs within this obesogenic context. Collectively, our data demonstrate that PA-induced Treg remodeling is contingent on the distinct VAT niche, which fosters a unique intrinsic transcriptional program in VAT Tregs.

### The ICOS^hi^ Treg subset regulates VAT inflammation and systemic metabolic homeostasis.

Although VAT Treg heterogeneity is well recognized for its adaptability to hormonal and growth factor signals (17, 18), the effect of obesity on their subset composition and functional specialization remains incompletely understood. In this study, we identified 2 distinct VAT Treg subsets, marked by high expression of ICOS or KLRG1, that are distinct from previously reported cell populations. Adoptive transfer of ICOS^hi^ Tregs into *Rag1^–/–^* mice alleviated VAT inflammation and improved systemic metabolic homeostasis more effectively than KLRG1^hi^ Tregs. Notably, the 2 subsets exhibited distinct functional preference: the ICOS^hi^ Treg subset displayed a predominant immunosuppressive capacity, particularly in IL-10 production, whereas KLRG1^hi^ Tregs were more engaged in regulating metabolic processes.

Interestingly, our findings are consistent with previous reports demonstrating subset‑specific IL‑10 production by Tregs across different tissues, including the c‑MAF (MAF bZIP transcription factor)–driven RORγ^+^ (retinoic acid‑related orphan receptor gamma) Treg subset in the colo (49), the PPARγ^+^ Treg population in the skin (38, 50, 51), and the functionally opposing IL‑10–producing versus nonproducing Treg subsets within the colorectal cancer microenvironment (52). Moreover, we found that *Crebzf* deletion amplified the IL-10 production in the ICOS^hi^ Treg subset, which was consistent with its enhanced immunosuppressive ability of ICOS^hi^ Tregs. Taken together, these data suggest that *Crebzf* deficiency may improve adipose tissue inflammation and metabolic status, enhance insulin sensitivity, and protect against diet‑induced obesity by promoting the accumulation and immunosuppressive function of ICOS^hi^ Tregs.

The paradigm of subset-specific regulation within the VAT Treg compartment is supported by prior work, which has delineated distinct transcriptional programs controlling major subsets. GATA3, PPARγ, and IL-33 serve as core regulators for ST2^+^ VAT Treg differentiation, whereas T-bet and IFN-γ are essential for CXCR3^+^ VAT Treg development (18). SREBP2-mediated cholesterol homeostasis enhances TCR signaling strength and preferentially promotes ST2^hi^ VAT Treg accumulation (19). Here, we establish CREBZF as a central regulator of VAT ICOS^hi^ Treg homeostasis, as its deletion rescues HFHS diet- or PA-driven loss of this subset and augments its suppressive capacity.

Treg subsets exhibit heterogeneous responses, adapting their functions to diverse tissue niches and disease states. Skin and VAT are the 2 major tissues enriched for PPARγ^+^ Tregs, which phenotypically share high expression of markers such as ST2 and KLRG1 and function to maintain tissue homeostasis. Conversely, KLRG1^+^ Tregs acquire senescent features characterized by mitochondrial dysfunction and genomic alterations, culminating in impaired suppressive activity and a proinflammatory phenotype during aging (53). In this context, our study adds a notable layer to the heterogeneity map of VAT Tregs by identifying CREBZF as an obesity-responsive regulator of the ICOS^hi^ Treg subset, a key immunomodulatory module that may represent adaptation to the metabolic stress of progressive obesity. Thus, the functional specialization of Treg subsets is shaped by their tissue niche and engagement in discrete physiological or pathological processes.

### CREBZF restricts the functional integrity of ICOS^hi^ Tregs.

Our study identifies PA-driven CREBZF overactivation as a pivotal factor that impairs ICOS^hi^ Tregs, thereby exacerbating VAT inflammation and systemic metabolic dysfunction. First, we found that CREBZF expression was higher in VAT T cells than in splenic T cells and was further upregulated in VAT Tregs during obesity. Second, genetic ablation of *Crebzf* selectively expanded and enhanced ICOS^hi^ Tregs in VAT, without affecting Treg homeostasis in other tissues or disrupting systemic immune balance. This spatial specificity positions CREBZF as a dedicated regulator of VAT ICOS^hi^ Tregs in obesity, rather than a universal Treg switch. Notably, the negative correlation between CREBZF and suppressive gene expression signatures in human VAT Tregs suggests a conservation of this regulatory axis in humans.

Current Treg-targeted strategies face 2 major challenges: precise tissue delivery and avoidance of systemic immune disruption. Consequently, niche-specific functional adaptations of Tregs in metabolic tissues have gained attention. As a transcription factor specifically enriched in VAT Tregs, particularly the ICOS^hi^ subset, CREBZF may represent a potential tissue-specific target for VAT-specific immunotherapy. Compared with broad Treg modulation or systemic metabolic interventions, targeting CREBZF could allow finer control of local immune balance in VAT, thereby minimizing systemic side effects. Thus, our work may provide therapeutic strategies for treating obesity and metabolic diseases.

In summary, this study identifies CREBZF as a subset- and tissue-specific regulator of ICOS^hi^ Tregs in VAT under obesogenic conditions. Mechanistically, PA upregulated CREBZF in ICOS^hi^ Tregs, which disrupted their stability and immunosuppressive function, thereby altering the local inflammatory landscape and ultimately aggravating systemic metabolic disturbance. This PA/CREBZF/ICOS^hi^ Treg subset axis bridges VAT inflammation with systemic metabolic dysregulation and represents a promising precision therapy target due to its dual specificity for tissue and Treg subsets.

## Methods

### Sex as a biological variable.

Human VAT samples from both male and female donors were used for gene expression correlation analyses. In vivo metabolic phenotyping studies and flow cytometric analyses of immune cells were performed using male mice only, as female mice on a HFHS diet are resistant to developing obesity and associated metabolic abnormalities.

Additional methods can be found in the Supplemental Methods.

### Animal model.

All mouse lines were maintained on a C57/BL6 background. Treg-specific *Crebzf*-KO (*Foxp3*-Cre *Crebzf^fl/fl^*) mice were generated by crossing floxed *Crebzf* mice (25) with *Foxp3*-Cre mice (also expressing the *Foxp3*-driven YFP reporter) as previously described (33), and WT littermates (*Foxp3*-Cre) were used as the control. T cell–specific *Crebzf*-KO (*Cd4*-Cre *Crebzf^fl/fl^*) mice were generated by crossing floxed *Crebzf* mice with *Cd4*-Cre mice as previously described (54), and WT littermates (*Crebzf^fl/fl^*) were used as the control. Mice were fed a HFHS diet (D12327, Research Diets) for 16 weeks.

### Human adipose tissue.

Adipose tissue samples were obtained from adult patients who had undergone bariatric surgery or elective abdominal surgery at the Nanjing Drum Tower Hospital Affiliated to Nanjing University Medical School (Nanjing, China) and the Affiliated Hospital of Southwest Medical University (Luzhou, China) (Supplemental Table 1). BMI was calculated as weight divided by height squared.

### Metabolic phenotyping.

For glucose tolerance tests (GTTs), mice were fasted for 16 hours followed by i.p. injection with glucose solution (1 g/kg body weight). For insulin tolerance tests (ITTs), mice were fasted for 6 hours and then i.p. injected with 1 U/kg insulin solution. Blood glucose levels were determined 0, 15, 30, 60, 90, and 120 minutes after injection of glucose or insulin. A triglycerides determination kit (Thermo Fisher Scientific), a cholesterol determination kit (Thermo Fisher Scientific), and an insulin ELISA kit (MilliporeSigma) were used for plasma, respectively, according to the manufacturers’ instructions. For body composition analysis, whole-body composition of live mice was analyzed using nuclear magnetic resonance (NMR) technology (EchoMRI). Mouse body weight was monitored weekly. Food intake was measured for individually housed mice every day for 7 days (55).

### Treg adoptive transfer model.

Mouse CD4^+^CD25^+^YFP^+^KLRG1^hi^ or ICOS^hi^ Tregs were sorted from spleens of *Foxp3*-Cre or *Foxp3*-Cre *Crebzf^fl/fl^* mice. Then, 3 × 10^5^ Tregs were adoptively transferred into *Rag1^–/–^* mice. Mice were fed a HFHS diet for 12 weeks, and tissues were harvested for further assays.

### H&E staining.

For H&E staining, adipose tissues from mice fed a chow or HFHS diet were fixed with 4% formalin for 48 hours and embedded in paraffin. Tissue sections (5 μm thick) were stained with H&E according to standard protocols.

### Immunofluorescence.

For immunofluorescence live-cell staining, cells were attached to glass coverslips coated with poly-d-lysine (for Jurkat cells and primary T cells) or not (for HEK293T cells) in the appropriate medium. Cells were washed with ice-cold PBS and fixed with 4% paraformaldehyde (PFA), and then the cells were permeabilized with 1% Triton X-100 for 5 minutes and blocked with 1% BSA (MilliporeSigma) for 30 minutes. Next, cells were incubated with a primary antibody (1:200 dilution) at 4°C overnight, followed by incubation with a secondary antibody (1:400 dilution) at 37°C for 60 minutes and DAPI staining for 10 minutes. For immunofluorescence staining of adipose tissue paraffin sections, WAT samples were washed with ice-cold PBS and fixed in 4% PFA at 4°C. After paraffin embedding, the samples were cut into 2–5 μm slices and dewaxed, rehydrated, and permeated. After being restored to room temperature, the sections of WAT were soaked in PBS for 10 minutes and then blocked by 1% BSA for 30 minutes. Next, cells were incubated with a primary antibody (1:200 dilution) at 4°C overnight, followed by incubation with a secondary antibody (1:400 dilution) at 37°C for 60 minutes and DAPI staining for 10 minutes. Coverslips were mounted, sealed with nail polish, and assessed by fluorescence microscopy. Images were acquired through ×63 oil objective lens with a Zeiss LSM880 confocal laser-scanning microscope.

### Flow cytometry.

For surface staining, cells isolated from mice or in vitro culture were directly stained with antibodies and/or fixable live/dead dye with 2% FBS and 1 mM EDTA at 4°C for 20 minutes. For transcription factor staining, cells prestained with surface markers were fixed and permeabilized in FOXP3/Transcription Factor Fixation/Permeabilization Buffer at 4°C for 30 minutes, washed once with FOXP3/Transcription Factor Permeabilization buffer, and stained with target markers in the FOXP3/Transcription Factor Permeabilization buffer at 4°C for 30 minutes. For CREBZF staining, cells were stained with anti-CREBZF followed by staining with a 594-labeled anti-rabbit secondary antibody. For intracellular cytokine analysis, cells were stimulated with PMA (50 ng/mL) and ionomycin (1 μg/mL) at 37°C for 3 hours in the presence of GolgiStop (BD BIosciences) before staining. After stimulation, cells were stained with surface markers and then fixed and permeabilized with Fixation/Permeabilization solution for 30 minutes followed by staining cytokines in the Perm/Wash buffer after washing. Antibodies for flow cytometry were used at a 1:200 dilution. Stained cells were analyzed on a CytoFLEX S (Beckman Coulter) or a CytoFLEX LX (Beckman Coulter) using CytExpert, and data were analyzed using FlowJo, version 10.

### Cell isolation.

Primary single-cell suspensions from spleens and lymph nodes were prepared by gently straining the tissue through a 40 μm cell strainer in PBS. For adipose T cell analyses, adipose tissues were collected in ice-cold PBS, minced, and digested using collagenase I (1 mg/mL, Worthington) in digestion buffer at 37**°**C for 40 minutes with vigorous shaking by hand every 10 minutes, and then EDTA (10 mM) was added and incubated at 37°C for additional 5 minutes. The cell suspension was passed through a 100 μm/70 μm cell strainer and centrifuged at 500*g* at 4°C for 10 minutes. After centrifugation, adipocytes form a white layer on top, while the stromal vascular fraction (SVF) form a red/white pellet on the bottom of the tube. The adipocytes and supernatant were gently aspirated and discarded, and then RBC lysis buffer was added to the SVF to eliminate red blood cells. The pelleted cells were resuspended in PBS and stained for flow cytometric analysis or sorting (56). Tregs were sorted from SVF pellets, and cells were further subjected to sequencing or in vitro assays.

### Cell culture and treatment.

The human HEK293T and Jurkat T cells were purchased from Cell Bank, Type Culture Collection Committee, Chinese Academy of Sciences (Shanghai, China). The HEK293T cells were cultured in DMEM containing 5.5 mM d-glucose,10% FBS, and 1% penicillin-streptomycin (P/S) as described previously (57), and cells were incubated in a humidified atmosphere of 5% CO_2_ at 37°C and passaged every 2 days by trypsinization. The Jurkat T cells and Tregs were cultured in advanced RPMI 1640 (Gibco/Invitrogen, Thermo Fisher Scientific) containing 10% FBS (Gibco, Thermo Fisher Scientific) and 1% P/S. Cells were transfected with plasmids for 24 hours, followed by treatment with PA, oleic acid, lactic acid, insulin and glucose for 24 hours.

### RNA isolation and quantitative real-time PCR analysis.

Tissues and cells were homogenized in TRIzol Reagent (Life Technologies, Thermo Fisher Scientific) to extract total RNAs according to the manufacturer’s protocol. Total RNAs were then reversely transcribed to cDNA using HiScript III RT SuperMix for qPCR (+gDNA wiper) (catalog R323-01, Vazyme). The resulting cDNA was subjected to real-time PCR with gene-specific primers in the presence of ChamQ SYBR qPCR Master Mix (High ROX Premixed) (catalog Q341-02, Vazyme) using the StepOnePlus Real-Time PCR System (Applied Biosystems) as described previously (29) (The oligonucleotide primers used are listed in Supplemental Table 2). The specificity of the PCR amplification was verified by analyzing the melting curve. Data were analyzed using the ΔΔCt threshold cycle method. The mRNA levels of genes were normalized to those of β-actin and presented as relative levels to the control.

### scRNA-seq (10X Genomics).

The scRNA-seq libraries were prepared following the protocol provided by 10X Genomics Chromium Single Cell Immune Profiling Solution. For Treg purification, CD4^+^CD25^+^YFP^+^ adipose tissue Tregs were FACS sorted from *Foxp3*-Cre or *Foxp3*-Cre *Crebzf^fl/fl^* mice fed a HFHS diet for 16 weeks. Dissociated cells were washed with 1× PBS containing 2% FBS. Cells were stained with 0.4% Trypan blue (Thermo Fisher Scientific, catalog 14190144) to check the viability on a Countess II Automated Cell Counter (Thermo Fisher Scientific). In each lane of a 10X Chromium instrument, approximately 10,000 adipose tissue Tregs were encapsulated into droplets. After reverse transcription, droplets were broken, and barcoded cDNA was purified with beads, followed by PCR amplification. All the remaining procedures including the library construction were performed according to the standard manufacturer’s protocol (Chromium Single Cell 3′ version 3). scRNA-seq libraries were quantified using a High Sensitivity DNA Chip (Agilent Technologies) on a Bioanalyzer 2100 and the Qubit High Sensitivity DNA Assay (Thermo Fisher Scientific). The libraries were sequenced on a NovaSeq 6000 (Illumina) using 2 × 150 chemistry. Raw data from all samples were analyzed in CellRanger software (10X Genomics, version 6.0.1) using “cellranger count” (CellRanger software), aligning the reads to the mouse reference genome (mm10). Filtering unique molecular identifiers (UMIs) with default parameters in CellRanger, the “filtered_feature_bc_matrix” was loaded using Seurat (version 3.2.0). After initial data loading and annotation, all Tregs were extracted from the combined samples. We then applied the SCTransform function to the extracted Tregs for normalization, scaling, and identification of 3,000 highly variable genes. Then, cells were integrated with the “IntegrateData” function. After reduction and clustering, we checked markers of Tregs and filtered small clusters with a high mitochondrion score. The Seurat pipeline was followed to find the clusters and create the UMAP plots. All DEGs were found using the “FindMarkers” function in Seurat with the parameters “logfc.threshold = 0.2, minutes.pct = 0.2.” To evaluate biological pathway activity, the AUCell package (version 1.16.0) was used to calculate ranks for SCTransform data on Tregs. All pathways were downloaded from Reactome (https://reactome.org/?ref=blog.opentargets.org). To infer the regulatory network of Treg subpopulations, we used the pySCENIC package (version 0.12.1) to explore the regulon of transcription factors. GO and Kyoto Encyclopedia of Genes and Genomes (KEGG) enrichment analyses were conducted with the clusterProfiler package (version 4.2.2). Other visualization images were generated by ggplot2 package (version 3.4.0).

### Statistics.

For clinical data, human correlations were performed using Pearson’s correlation analysis. Statistical significance between gene expression levels was evaluated by an unpaired, 2-tailed Student’s *t* test. For animal and in vitro studies, data are expressed as the mean ± SEM, and statistical significance was evaluated by unpaired, 2-tailed Student’s *t* test for comparison of 2 groups. For more than 2 groups, data were analyzed by 1- or 2-way ANOVA. Statistical analyses were performed using statistical tools in GraphPad Prism 8.0 (GraphPad Software). Differences were defined as significant at a *P* value of less than 0.05.

### Study approval.

All mice were housed under a 12-hour light/12-hour dark cycle under a controlled temperature. All animal experimental protocols were approved by the IACUC of the Shanghai Institute of Nutrition and Health, Chinese Academy of Sciences (SINH-2025-LY-1). The study protocol was approved by the Ethics Review Committee of the Nanjing Drum Tower Hospital Affiliated to Nanjing University Medical School and the Affiliated Hospital of Southwest Medical University and was conducted in accordance with the 1975 Declaration of Helsinki.

### Data and code availability.

All of our expression data have been deposited in National Omics Data Encyclopedia (https://www.biosino.org/node) under the accession number OEP00005631. As for RNA-seq, the expression of *Crebzf* in VAT Tregs was profiled using the published dataset GSE174706 from the Gene Expression Omnibus (GEO) database. All other data supporting the findings of this study are available from the corresponding authors upon reasonable request. Source data are provided with this work. Data points can be accessed from the Supporting Data Values file.

## Author contributions

WS, Y Liu, XY, AC, and Y Li contributed to experimental design. WS, Y Liu, XY, MH, LX, JL, XC, PH, JW, DD, ZZ, WL, LL, ZL, KQ, JG, and HL contributed to the acquisition and analysis of data. CG, HW, TZ, XM, WL, BL, HZ, and YB provided reagents and material support. BL, YB, and CZ edited the manuscript with important intellectual content. Y Li, CZ, YB, AC, and WS obtained the funding. WS and Y Li wrote the manuscript.

## Conflict of interest

The authors have declared that no conflict of interest exists.

## Funding support

- Noncommunicable Chronic Diseases-National Science and Technology Major Project (2024ZD0531300, to YL).
- Strategic Priority Research Program of the Chinese Academy of Sciences (XDB1150000, to YL).
- National Key R&D Program of China (2023YFA1801100, 2019YFA0802502, to YL).
- National Natural Science Foundation of China (32130047, to YL; 82030007, U23A20398, to CZ; 82450002, 82030026, 82270883, to YB; 32371206, 32100943, to AC).
- Postdoctoral Fellowship Program of CPSF (GZC20251002, to WS).
- Shanghai Municipal Science and Technology Major Project (to YL).
- Natural Science Foundation of Shanghai (24ZR1478200, to WS).
- Sichuan Science and Technology Program (2024YFFK0081, to YL).
- Open Project Program of Metabolic Vascular Diseases Key Laboratory of Sichuan Province (2024MVDKL-K1, to YL; 2023MVDKL-K2, to AC; 2025MVDKL-K3, to WS).

## Supplementary Material

Supplemental data

Unedited blot and gel images

Supporting data values

## Version 1 07/23/2026

In-Press Preview

## Version 2 09/15/2026

Electronic publication
