# Supplementary material for: Palmitic acid coordinates impaired visceral adipose ICOShi Treg-mediated immunosuppression and systemic metabolic disturbance during obesity
Source: J Clin Invest. 2026 Jul 23;136(18):e207089. doi: 10.1172/JCI207089 (PMC13574151; doi:10.1172/JCI207089)
Supplement: Supplementary file 1 [file jci-136-207089-s042.pdf]

Palmitic acid coordinates impaired visceral adipose ICOS^{hi} Treg-mediated immunosuppression and systemic metabolic disturbance during obesity

Weitong Su[1,8], Yuxiao Liu[1,8], Xi Yan[1,8], Mengyao Huang[1], Linghao Xu[2], Jing Lin[1], Xufeng Chen[1], Puyuan Hu[1], Chenlin Gao[2], Jian Wen[3,4], Hongdong Wang[5], Dong Ding[1], Zengpeng Zheng[1], Wenjing Li[1], Lianjia Li[1], Zhan Liu[1], Keyu Qian[1], Jing Gao[1], Tingting Zhang[1], Xiaobing Mao[2], Haibing Zhang[1], Wei Lu[1], Bin Li[6], Hong Li[1], Aoyuan Cui[1]*, Yan Bi[5]*, Chunxiang Zhang[7]*, Yu Li[1]*

[1]Shanghai Institute of Nutrition and Health, University of Chinese Academy of Sciences, Chinese Academy of Sciences, Shanghai 200031, China; [2]Department of Endocrinology and Metabolism, Metabolic Vascular Disease Key Laboratory of Sichuan Province, The Affiliated Hospital of Southwest Medical University, Luzhou, Sichuan 646000, China; [3]Department of General Surgery (Hepatobiliary Surgery), Biliary-Pancreatic Center, The Affiliated Hospital, Southwest Medical University, Luzhou, Sichuan 646000, China; [4]Metabolic Hepatobiliary and Pancreatic Diseases Key Laboratory of Luzhou City, Academician (Expert) Workstation of Sichuan Province, The Affiliated Hospital, Southwest Medical University, Luzhou, Sichuan 646000, China; [5]Department of Endocrinology, Endocrine and Metabolic Disease Medical Center, Nanjing Drum Tower Hospital, Affiliated Hospital of Medical School, Nanjing University, Nanjing 210008, China; [6]Department of Immunology and Microbiology, Shanghai Institute of Immunology, Shanghai Jiao Tong University School of Medicine, Shanghai 200025, China; [7]Metabolic Vascular Disease Key Laboratory of Sichuan Province, The Affiliated Hospital of Southwest Medical University, Key Laboratory of Medical Electrophysiology, Ministry of Education, Southwest Medical University, Luzhou, 646000, China; [8]These authors contribute equally to this work.

Running Title: Palmitic acid impairs ICOS^{high} Treg cells in visceral adipose tissue

Supplemental Figure Legends

Fig.S1. CREBZF is highly expressed in T cells. (A) Human *CREBZF* expression in different cell types (left) and clusters (middle) identified in the vascular and visualized by a UMAP plot, as well as a bar chart (right). The T cell clusters were highlighted in red color. (B) Human *CREBZF* gene expression in multiple tissues from the Human Protein Atlas (https://www.proteinatlas.org/tissue). (C) Human *CREBZF* gene expression in immune cell types from the Human Protein Atlas (https://www.proteinatlas.org/immune cell). (D) mRNA levels of *Crebzf* in different tissues of mice. Data were presented as mean ± SEM. n = 6. (E) Pearson correlation of *CREBZF* mRNA expression in human VAT with HOMA-IR and plasma insulin levels. n = 33. (F) Pearson correlation of *CREBZF* mRNA expression with conventional T cell marker genes in human subcutaneous adipose tissue (SAT). n = 57. (G) Representative immunofluorescence images of $CD3^{+}$ T cells in VAT from lean and obese subjects. (H) Quantification of CREBZF immunofluorescence intensity. Data were presented as mean ± SEM. n = 6-7. 2-tailed unpaired Student's t test. *p < 0.05 vs. lean.

Fig.S2. T cell-specific *Crebzf* deficiency increases systemic energy expenditure in HFHS diet-fed mice. (A) Genotypes of wide-type (WT) (+/+), *Crebzf* heterozygous (+/-), and T cell *Crebzf* knockout (-/-) mice were determined by PCR amplification. Floxed *Crebzf*, WT, and *Cd4*-Cre alleles were evidenced by amplification of 318-bp, 248-bp, and 265-bp fragments, respectively. (B) Relative mRNA levels of *Crebzf* in $CD3^{+}$ T cells, $CD19^{+}$ B cells, $CD4^{+}$ T cells, and $CD8^{+}$ T cells sorted from spleen and lymph nodes of $Crebzf^{fl/fl}$ and *Cd4*-Cre $Crebzf^{fl/fl}$ mice. n = 3-4. (C) Expression levels of *Crebzf* in $CD3^{+}$ T cells, $CD19^{+}$ B cells, $CD4^{+}$ T cells, and $CD8^{+}$ T cells were measured by immunoblots. (D) Representative appearance of liver from $Crebzf^{fl/fl}$ and *Cd4*-Cre $Crebzf^{fl/fl}$ mice fed with chow or HFHS diet. (E) Plot of body composition showing the absolute amount of fat, lean, and total mass (g) in $Crebzf^{fl/fl}$ and *Cd4*-Cre $Crebz^{fl/fl}$ mice fed with chow diet. n = 8. (F) Tissue weights of liver, eWAT, iWAT, BAT, and spleen in $Crebzf^{fl/fl}$ and *Cd4*-Cre $Crebzf^{fl/fl}$ mice fed with chow diet. n = 5-7. (G) The ratios of tissue weights of liver, eWAT, iWAT, and BAT to body weight in $Crebzf^{fl/fl}$ and *Cd4*-Cre $Crebzf^{fl/fl}$ mice fed with chow diet. n = 5-7. (H) Plot of body composition showing the absolute amount of fat, lean, and total mass (g) in $Crebzf^{fl/fl}$ and *Cd4*-Cre $Crebzf^{fl/fl}$ mice fed with HFHS diet. n = 22-23. (I) Tissue weights of liver, eWAT, iWAT, BAT, and spleen in $Crebzf^{fl/fl}$ and *Cd4*-Cre $Crebzf^{fl/fl}$ mice fed

with HFHS diet. n = 7-17. (J) The ratios of tissue weights of liver, eWAT, iWAT, and BAT to body weight in *Crebzf*$^{fl/fl}$ and *Cd4*-Cre *Crebzf*$^{fl/fl}$ mice fed with HFHS diet. n = 7-17. (K) Body weight curves of the mice fed with HFHS diet. n = 19-20. (L) Food intake of *Crebz*$^{fl/fl}$ and *Cd4*-Cre *Crebzf*$^{fl/fl}$ mice fed with chow or HFHS diet. n = 7-11. (B-L) Data were presented as mean ± SEM. 2-tailed unpaired Student's t test. *p < 0.05, vs. *Crebzf*$^{fl/fl}$.

Fig.S3. CREBZF specifically regulates Treg cell homeostasis in epididymal adipose tissue. (A and B) The frequencies (A) and numbers (B) of $CD4^+$ Th1 cells in eWAT from *Crebzf*$^{fl/fl}$ and *Cd4*-Cre *Crebzf*$^{fl/fl}$ mice fed with chow or HFHS diet. (C and D) The frequencies (C) and numbers (D) of $CD4^+$ Th17 cells in eWAT from *Crebzf*$^{fl/fl}$ and *Cd4*-Cre *Crebzf*$^{fl/fl}$ mice fed with chow or HFHS diet. (A-D) Data were presented as mean ± SEM. Chow diet group, n = 4; HFHS diet group, n = 3-5. Two-way ANOVA for multiple comparison. *p < 0.05 vs. *Crebzf*$^{fl/fl}$ and chow. (E and F) Representative flow cytometric plots (E) and frequencies (F) of $CD25^+$ $FOXP3^+$ Treg cells in spleen, lymph nodes, thymus, blood, inguinal white adipose tissue (iWAT), liver, small intestinal lamina propria (SI-LP), and lung from *Crebzf*$^{fl/fl}$ and *Cd4*-Cre *Crebzf*$^{fl/fl}$ mice fed with chow or HFHS diet. Chow diet group, n = 5-6; HFHS diet group, n = 4-6. 2-tailed unpaired Student's t test.

Fig.S4. Comparable body composition and energy expenditure in wild type and Treg cell *Crebzf*-deficient mice. (A) Genotypes of wide-type (WT) (+/+), *Crebzf* heterozygous (+/-), and Treg cell *Crebzf* knockout (-/-) mice were determined by PCR amplification. Floxed *Crebzf*, WT, and *Foxp3*-Cre WT and mutant alleles were evidenced by amplification of 318-bp, 248-bp, 630-bp, and 346-bp fragments, respectively. (B) Relative mRNA levels of *Crebzf* in Treg and Tconv cells sorted from spleen and lymph nodes of *Foxp3*-Cre and *Foxp3*-Cre *Crebzf*$^{fl/fl}$ mice. n = 3. (C) Representative histograms (left) and MFI (right) of CREBZF in $CD4^+$ YFP^+ Treg and $CD4^+$ $CD25^-$ YFP^- Tconv cells. n = 6-10. (D) Representative appearance of liver form *Foxp3*-Cre and *Foxp3*-Cre *Crebzf*$^{fl/fl}$ mice fed with chow or HFHS diet. (E) Plot of body composition showing the absolute amount of fat, lean, and total mass (g) in *Foxp3*-Cre and *Foxp3*-Cre *Crebzf*$^{fl/fl}$ mice fed with chow diet. n = 6. (F) Tissue weights of liver, eWAT, iWAT, BAT, and spleen in *Foxp3*-Cre and *Foxp3*-Cre *Crebzf*$^{fl/fl}$ mice fed with chow diet. n = 8-9. (G) The ratios of tissue weights of liver, eWAT, iWAT, and BAT to body weight in *Foxp3*-Cre and *Foxp3*-Cre *Crebzf*$^{fl/fl}$ mice fed with chow diet. n = 8-9. (H) Plot of body composition showing the

absolute amount of fat, lean, and total mass (g) in *Foxp3*-Cre and *Foxp3*-Cre *Crebzf*$^{fl/fl}$ mice fed with HFHS diet. n = 13. (I) Tissue weights of liver, eWAT, iWAT, BAT, and spleen in *Foxp3*-Cre and *Foxp3*-Cre *Crebzf*$^{fl/fl}$ mice fed with HFHS diet. n = 12-18. (J) The ratios of tissue weights of liver, eWAT, iWAT, and BAT to body weight in *Foxp3*-Cre and *Foxp3*-Cre *Crebzf*$^{fl/fl}$ mice fed with HFHS diet. n = 12-18. (K) Body weight curves of the *Foxp3*-Cre and *Foxp3*-Cre *Crebzf*$^{fl/fl}$ mice fed with HFHS diet. Data were presented as mean ± SEM. n = 7-8. (L) Food intake of *Foxp3*-Cre and *Foxp3*-Cre *Crebzf*$^{fl/fl}$ mice fed with chow or HFHS diet. n = 10-11. (B-L) Data were presented as mean ± SEM. 2-tailed unpaired Student's t test. *p < 0.05, vs. *Foxp3*-Cre.

Fig.S5. *Crebzf* deficiency does not alter Treg cell frequencies in multiple tissues. (A and B) The frequencies (A) and numbers (B) of CD3$^+$ T cells in eWAT from *Foxp3*-Cre and *Foxp3*-Cre *Crebzf*$^{fl/fl}$ mice fed with chow or HFHS diet. (C and D) The frequencies (C) and numbers (D) of CD4$^+$ T cells in eWAT from *Foxp3*-Cre and *Foxp3*-Cre *Crebzf*$^{fl/fl}$ mice fed with chow or HFHS diet. (E and F) The frequencies (E) and numbers (F) of CD8$^+$ T cells in eWAT from *Foxp3*-Cre and *Foxp3*-Cre *Crebzf*$^{fl/fl}$ mice fed with chow or HFHS diet. (G and H) The frequencies of total macrophages (G) and TREM2$^+$ macrophages (H) in eWAT from *Foxp3*-Cre and *Foxp3*-Cre *Crebzf*$^{fl/fl}$ mice fed with chow or HFHS diet. (A-H) Data were presented as mean ± SEM. Chow diet group, n = 3-6; HFHS diet group, n = 4-8. Two-way ANOVA for multiple comparison. *p < 0.05 vs. *Foxp3*-Cre and chow; #p < 0.05, vs. *Foxp3*-Cre and HFHS. (I and J) Representative flow cytometric plots (I) and frequencies (J) of FOXP3$^+$ Treg cells in spleen, lymph nodes, thymus, blood, iWAT, liver, small intestinal lamina propria (SI-LP), and lung from *Foxp3*-Cre and *Foxp3*-Cre *Crebz*$^{fl/fl}$ mice fed with chow or HFHS diet. Data were presented as mean ± SEM. Chow diet group, n = 3-6; HFHS diet group, n = 3-9. 2-tailed unpaired Student's t test.

Fig. S6. T cell activation does not affect CREBZF expression. (A) The mRNA levels of *CREBZF* in Jurkat T cells treated with palmitic acid or vehicle for 24h as indicated. n = 6. (B) RNA-seq quantification of *Crebzf* transcript levels under HFD conditions at different time points in GSE174706 dataset. (C-E) Jurkat T cells were stimulated with 100 ng/ml PMA and 1 μg/ml ionomycin for the indicated time course. The mRNA levels of *CREBZF* in Jurkat T cells were shown in C (short term), D (middle term), and E (long term). (F-H) CD3$^+$ T cells were isolated from spleen and lymph nodes of

mice and stimulated with 2 μg/ml anti-CD3 and 1 μg/ml CD28 antibody for the indicated time course. The mRNA levels of *Crebzf* in $CD3^+$ T cells were shown in F (short term), G (middle term), and H (long term). (A-H) Data were presented as mean ± SEM. n = 3-6. One-way ANOVA for multiple comparison. *p < 0.05 vs. vehicle. (I) Body weights of the *Foxp3*-Cre *Crebzf*$^{fl/fl}$ and control *Foxp3*-Cre mice received daily intraperitoneal injections of palmitic acid (ethyl palmitate, 3 μM/g) or vehicle. Data were presented as mean ± SEM. n = 3-4. Two-way ANOVA for multiple comparison.

Fig.S7. *Crebzf* deficiency enhances the suppressive function of ICOS^{hi} Treg. (A) Heatmap of differentially expressed genes of Treg subpopulations. (B) Violin plots for other top markers of Treg subpopulations. (C) The frequencies of KLRG1^{hi} and ICOS^{hi} Treg cells in eWAT from *Foxp3*-Cre mice fed with chow or HFHS diet. Data were presented as mean ± SEM. n = 4. *p < 0.05 vs. chow. (D) Volcano plots comparing transcriptomes of ICOS^{hi} Treg cells from *Foxp3*-Cre *Crebzf*$^{fl/fl}$ versus *Foxp3*-Cre mice. (E) Gene Ontology (GO) enrichment terms associated with DEGs of ICOS^{hi} Treg cells from *Foxp3*-Cre *Crebzf*$^{fl/fl}$ and *Foxp3*-Cre mice. (F) The numbers of ICOS^{hi} and KLRG1^{hi} Treg cells in eWAT from *Foxp3*-Cre and *Foxp3*-Cre *Crebzf*$^{fl/fl}$ mice fed with HFHS diet. n = 9. (G-I) Representative flow cytometric plots (G), frequencies (H), and numbers (I) of ICOS^{hi} and KLRG1^{hi} Treg cells in iWAT from *Foxp3*-Cre and *Foxp3*-Cre *Crebzf*$^{fl/fl}$ mice fed with HFHS diet. n = 5-6. (J-L) Representative flow cytometric plots (J), frequencies (K) and numbers (L) of ICOS^{hi} and KLRG1^{hi} Treg cells in spleen from *Foxp3*-Cre and *Foxp3*-Cre *Crebzf*$^{fl/fl}$ mice fed with HFHS diet. n = 3-5. (G-L) Data were presented as mean ± SEM. 2-tailed unpaired Student's t test.

Fig.S8. Improvement of obesity increases the proportion of ICOS^{hi} Treg cells in adipose tissue. (A) UMAP plots of immune cell subclusters from SAT of obese humans and humans after weight loss (left), and the populations (middle) and number (right) of Treg cells (PMID 41526584). (B) UMAP plots of immune cell subclusters from eWAT of obese mice and mice subjected to dietary switch from HFD to chow (weight loss) (left), and the populations (middle) and number (right) of Treg cells (PMID 41526584). (C) UMAP plots of immune cell subclusters from eWAT of obese mice and caloric restriction-induced weight loss mice (left), and the populations (middle) and number (right) of Treg cells (PMID 40627456). (D) Dot plot of Klrg1 and Icos genes for KLRG1^{hi} and ICOS^{hi} Treg cell subsets.

(E) Cluster composition of ICOS^{hi} and KLRG1^{hi} Treg cells in obese and caloric restriction-induced weight loss mice.

Fig.S9. CREBZF interacts with c-JUN to suppress *FOXP3* transcriptional activity in ICOS^{hi} Treg cells. (A-C) *Crebzf* deficiency enhances transcriptional co-regulator activity in epididymal adipose tissue Treg cells. (A) Heatmaps showing density of mapped ATAC-seq reads from single biological replicates 3 kb up- and downstream of transcriptional start sites (TSS). (B) Integrative Genomics Viewer (IGV) plot showed the ATAC-seq peaks on *Icos*, *Tigit*, *Klrg1*, and *Pparg* gene locus. (C) Top 10 enriched transcription factor recognition sequences in ATAC-seq peaks of eWAT Treg cells. (D) Transcription factor activities of ICOS^{hi} Treg cells in eWAT from *Foxp3*-Cre and *Foxp3*-Cre *Crebzf*$^{fl/fl}$ mice. (E-G) CREBZF associates with c-JUN. (E) Co-immunoprecipitation experiments were performed to detect transcription factors that interact with CREBZF. (F) myc-CREBZF and FLAG-c-JUN were transfected into HEK293T cells and followed by co-immunoprecipitation of FLAG. (G) Colocalization of CREBZF with c-JUN in Treg cells. (H) CREBZF inhibited the interaction of c-JUN with c-FOS. HEK293T cells were transfected with GST-tagged CREBZF, FLAG- tagged c-FOS and myc-tagged c-JUN followed by co-immunoprecipitation of anti-FLAG. (I) CREBZF diminished the transcriptional activity of *FOXP3*. HEK293T cells were transiently transfected with *FOXP3* promoter and Renilla luciferase reporter plasmid pRL-SV40, together with CREBZF, c-JUN, c-FOS or pcDNA as indicated. Dual luciferase activities were measured. Data were presented as mean ± SEM. n = 3. One-way ANOVA for multiple comparison. *p < 0.05 vs. pcDNA. #p < 0.05 vs. c-JUN and c-FOS. (J) The effects of CREBZF on inhibition of *Foxp3*, *Il10*, and *Tgfb* were abolished by knockdown of *c-Jun* in ICOS^{hi} Treg cells. ICOS^{hi} Treg cells were sorted and transfected with si*c-Jun* or siNC, followed by treatment with Rv-GFP or Rv-*Crebzf*. Transcriptional levels of *Foxp3*, *Il10*, and *Tgfb* were detected by qPCR. Data were presented as mean ± SEM. n = 3. Two-way ANOVA for multiple comparison. *p < 0.05 vs. siNC and Rv-GFP. (K) The mRNA levels of *c-Jun* in ICOS^{hi} Treg cells. Data were presented as mean ± SEM. n = 9. 2-tailed unpaired Student's t test. *p < 0.05 vs. siNC.

Fig.S10. VAT-specific inverse correlation of CREBZF with Treg suppressive genes. (A and B) Flow cytometric analysis of CREBZF and FOXP3 expression in Treg cells isolated from VAT of obese subjects (A) and from eWAT of mice (B). n = 9-12. (C) Correlation of *CREBZF* expression with *FOXP3*,

IL-10, and *TGFB* in SAT of humans. n = 57. (D) Correlation of *Crebzf* expression with *Foxp3*, *Il10*, and *Tgfb* in iWAT of mice. n = 45.

Supplementary Fig. S1

Su W, Liu Y, Yan X, et al.

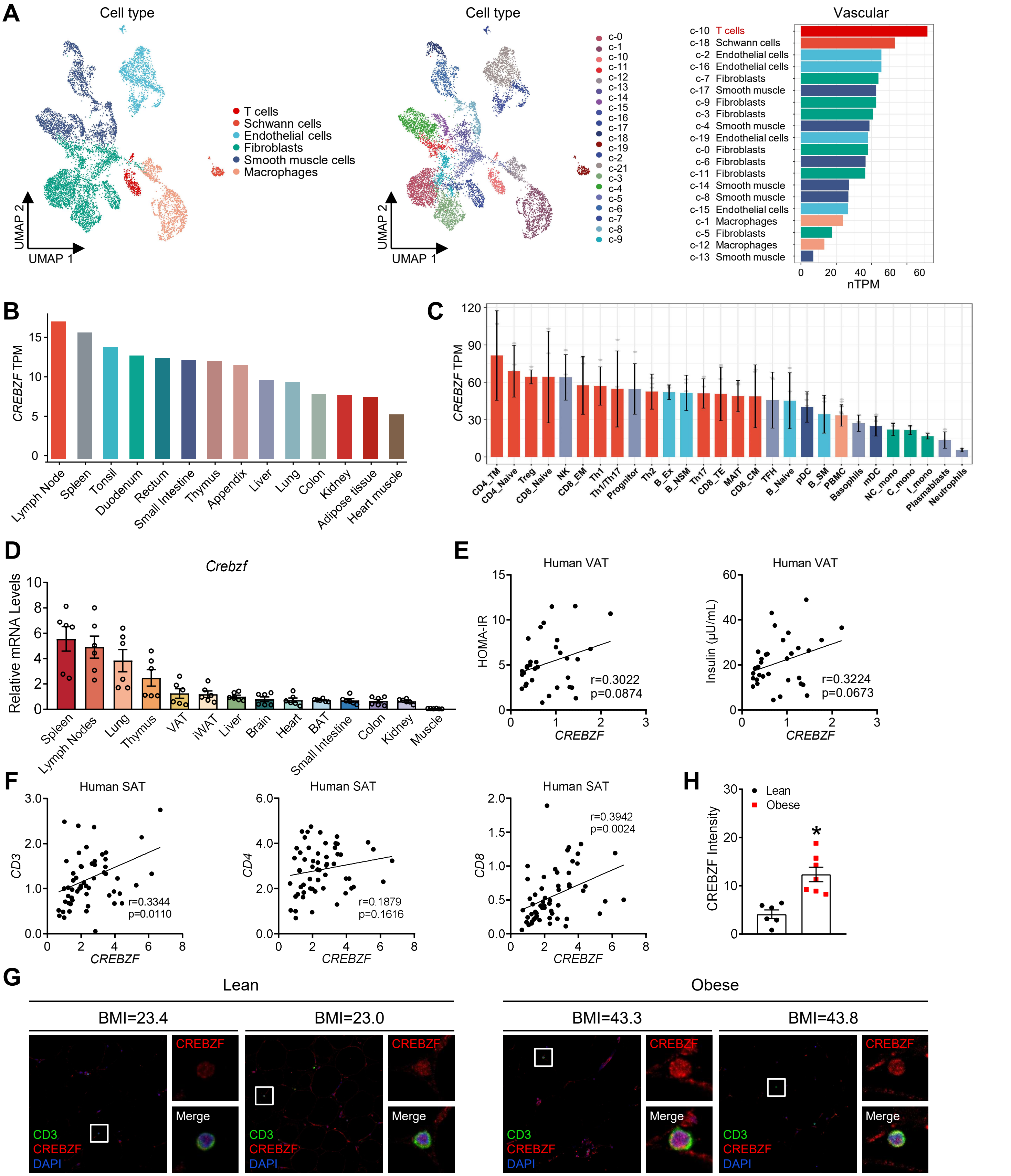

Supplementary Fig. S2

Su W, Liu Y, Yan X, et al.

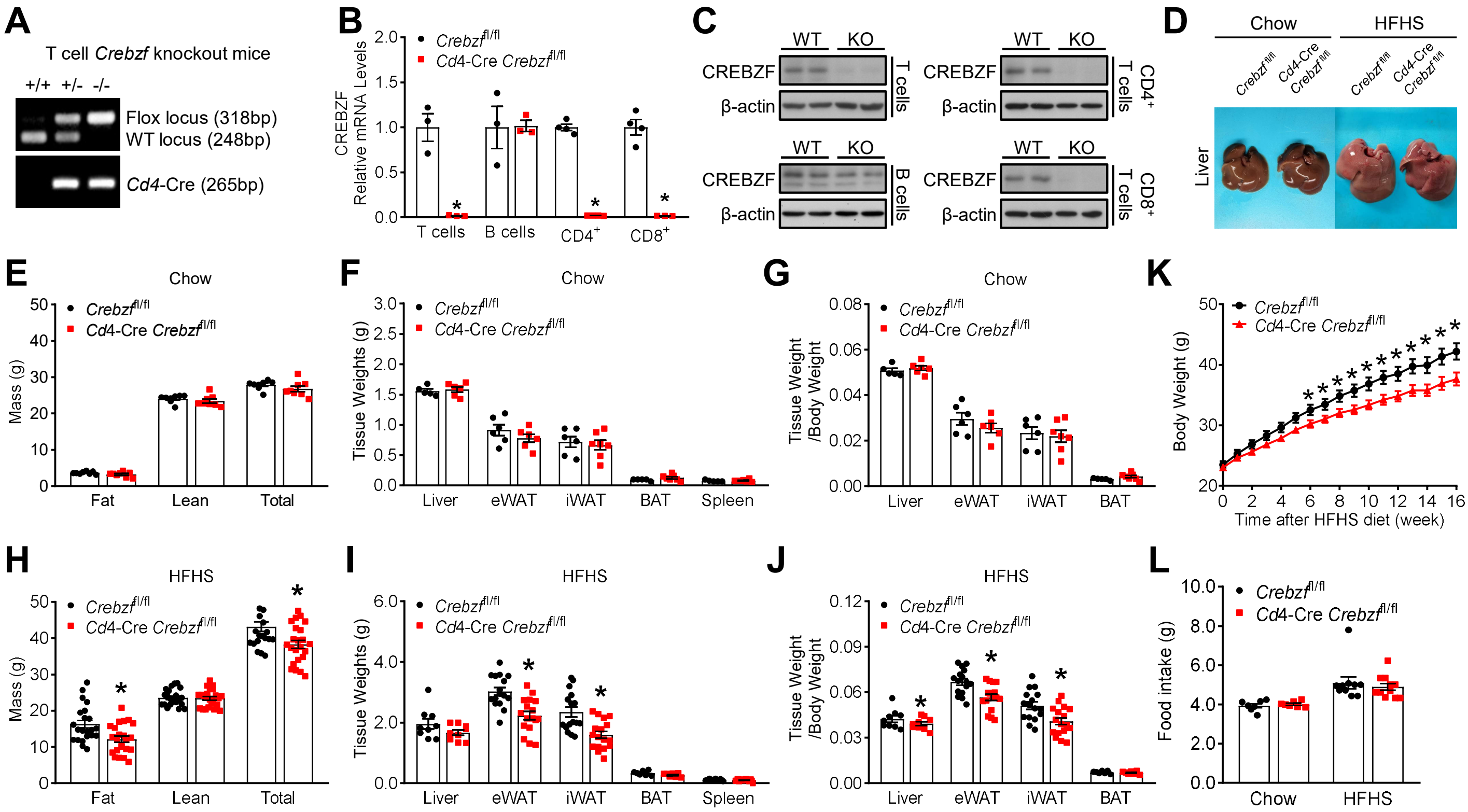

Supplementary Fig. S3

Su W, Liu Y, Yan X, et al.

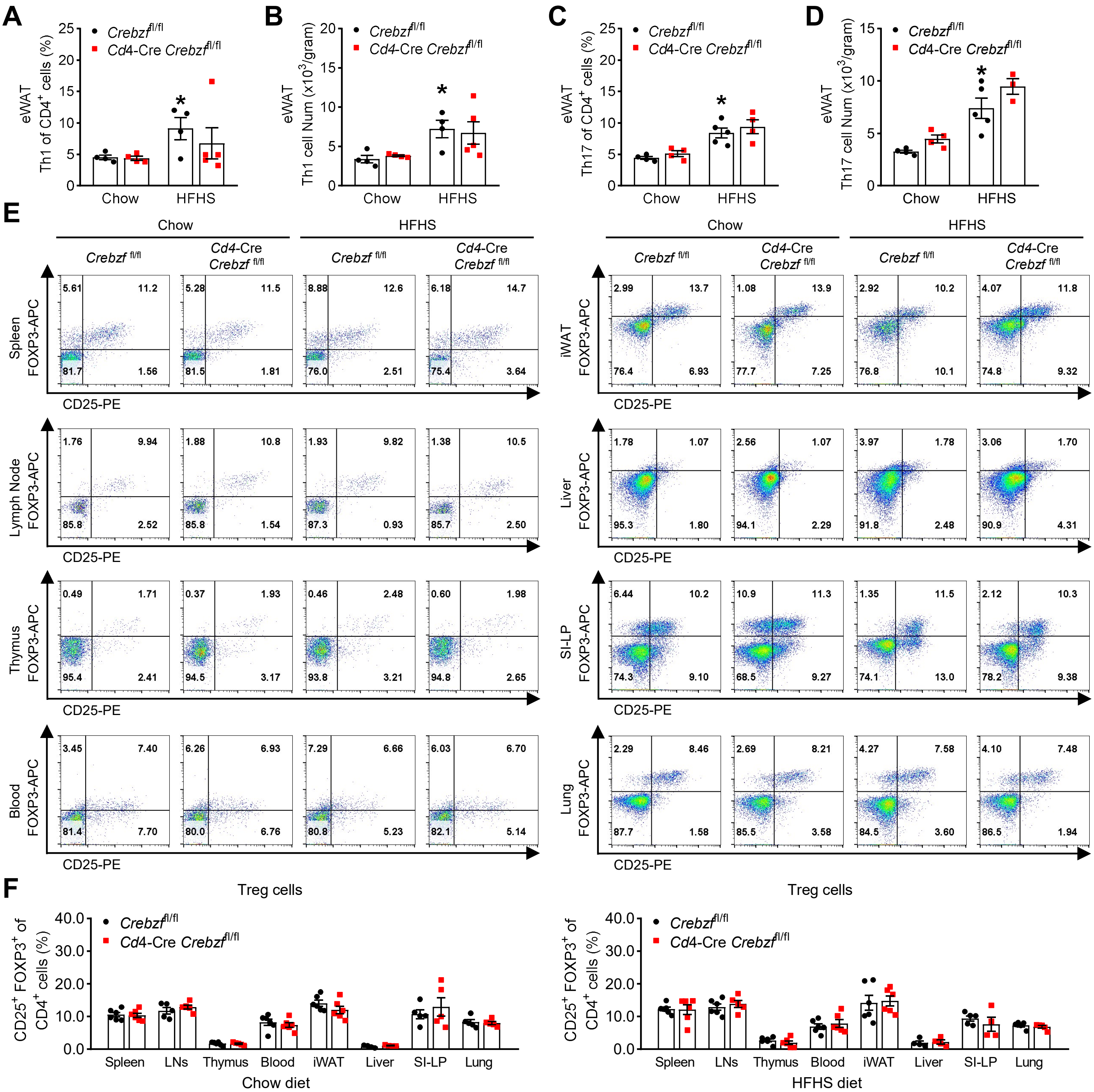

Supplementary Fig. S4

Su W, Liu Y, Yan X, et al.

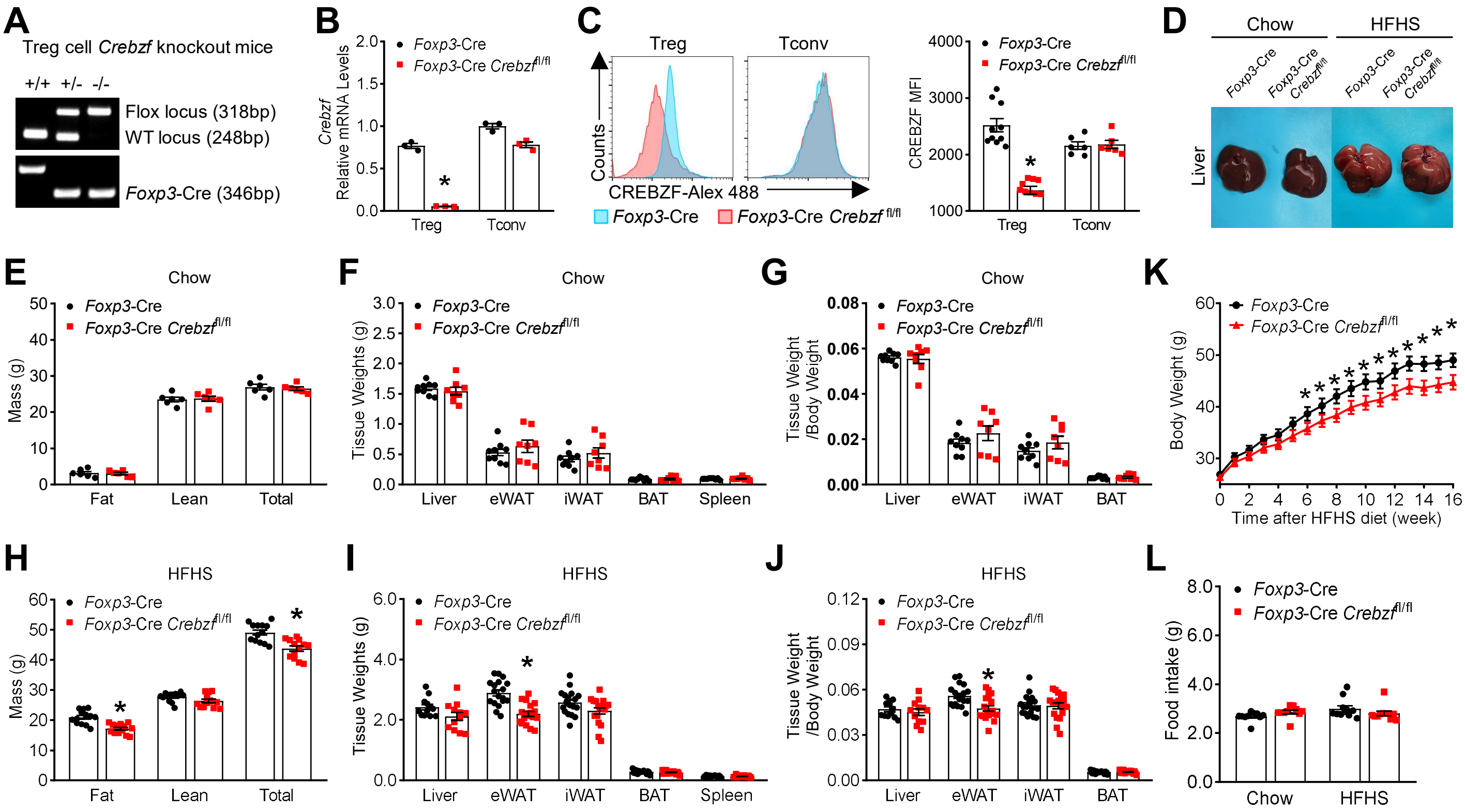

Supplementary Fig. S5

Su W, Liu Y, Yan X, et al.

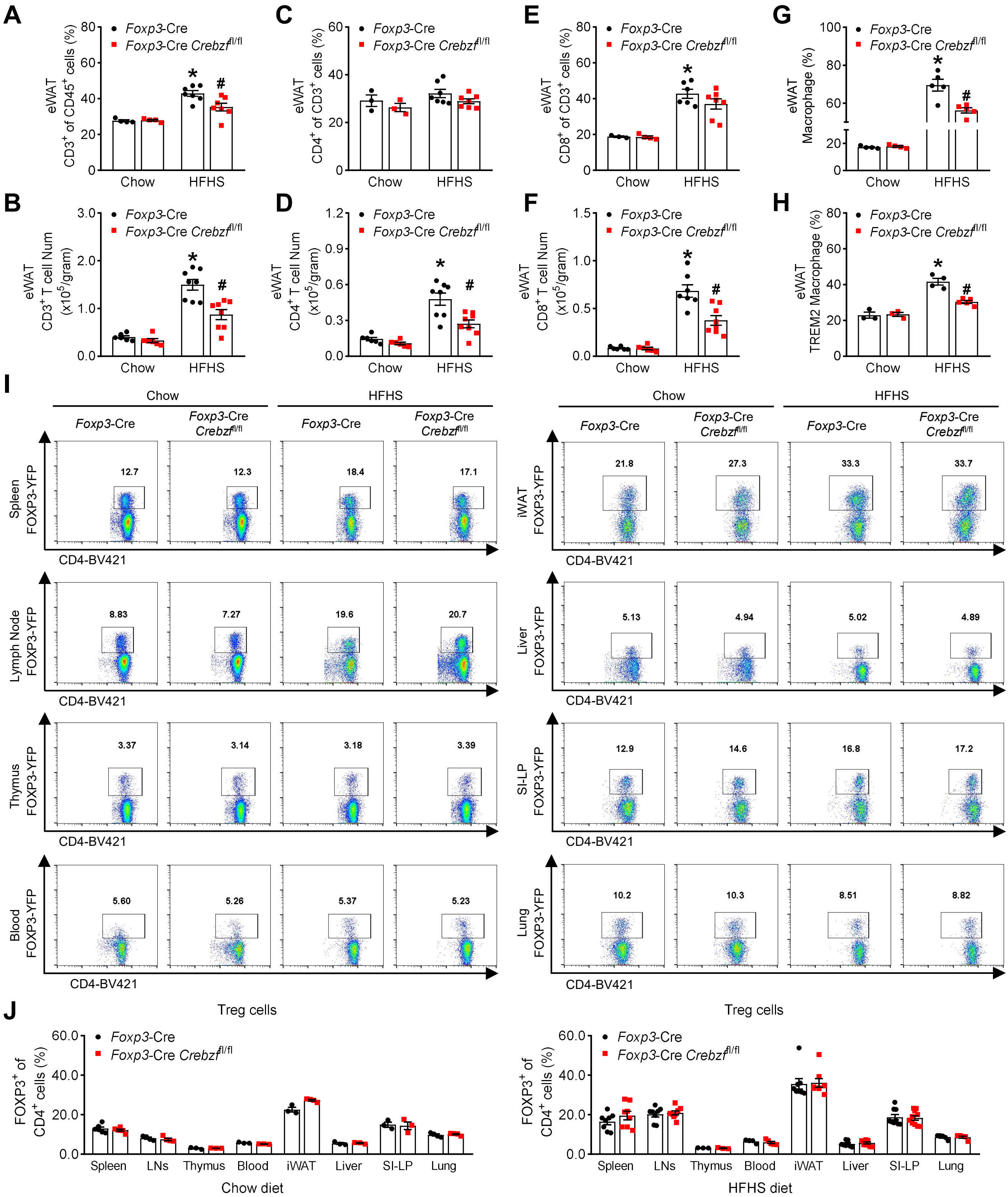

Supplementary Fig. S6

Su W, Liu Y, Yan X, et al.

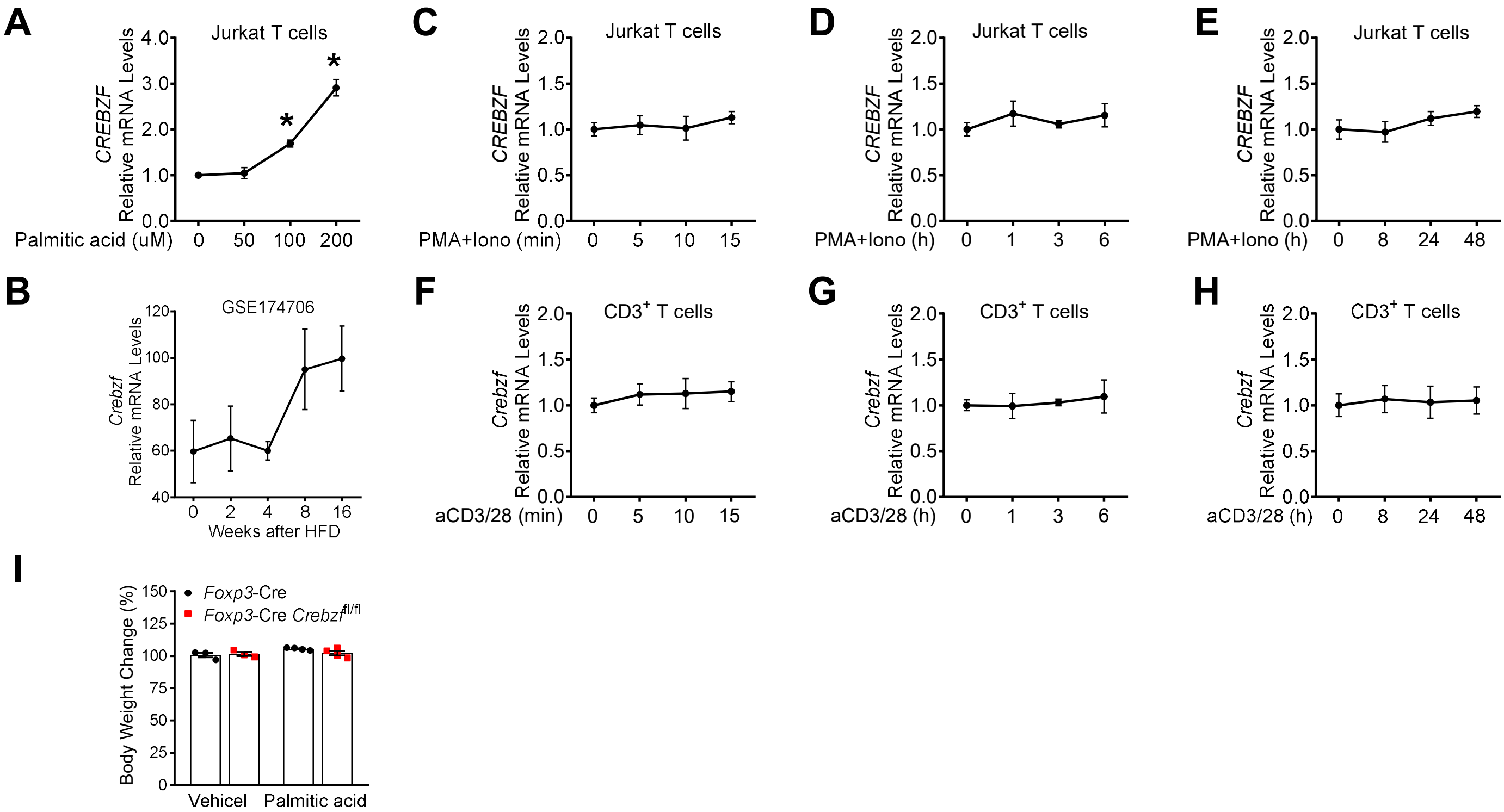

Supplementary Fig. S7

Su W, Liu Y, Yan X, et al.

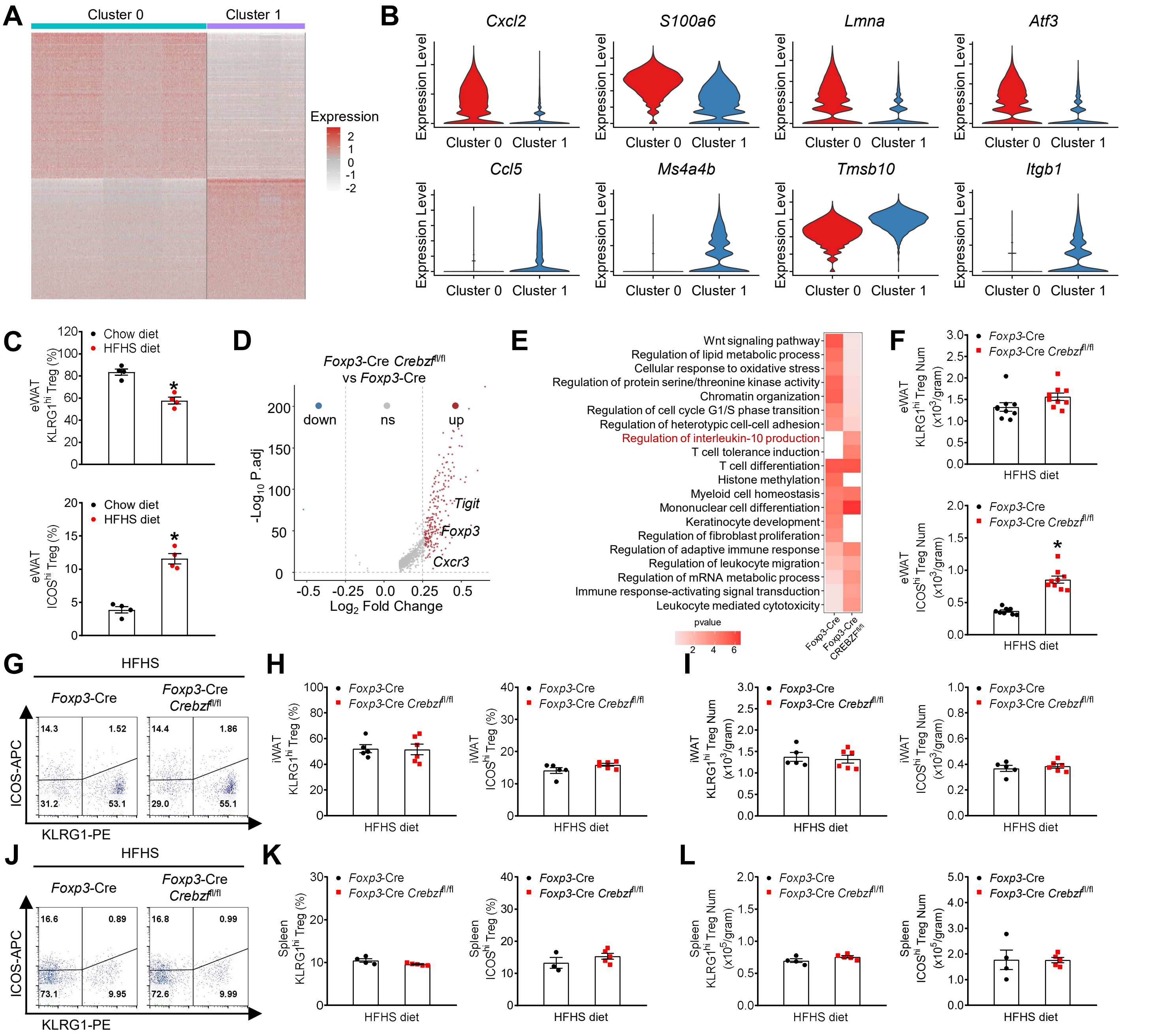

Supplementary Fig. S8

Su W, Liu Y, Yan X, et al.

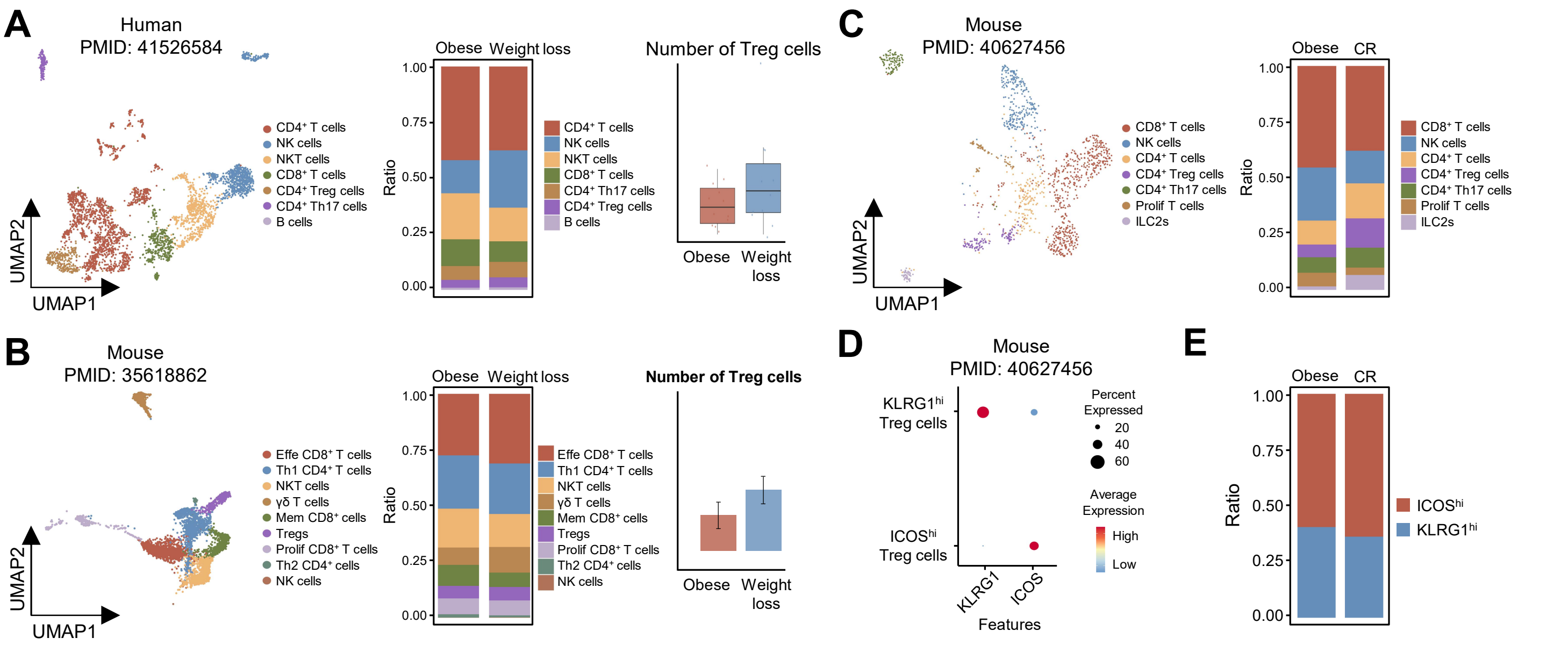

Supplementary Fig. S9

Su W, Liu Y, Yan X, et al.

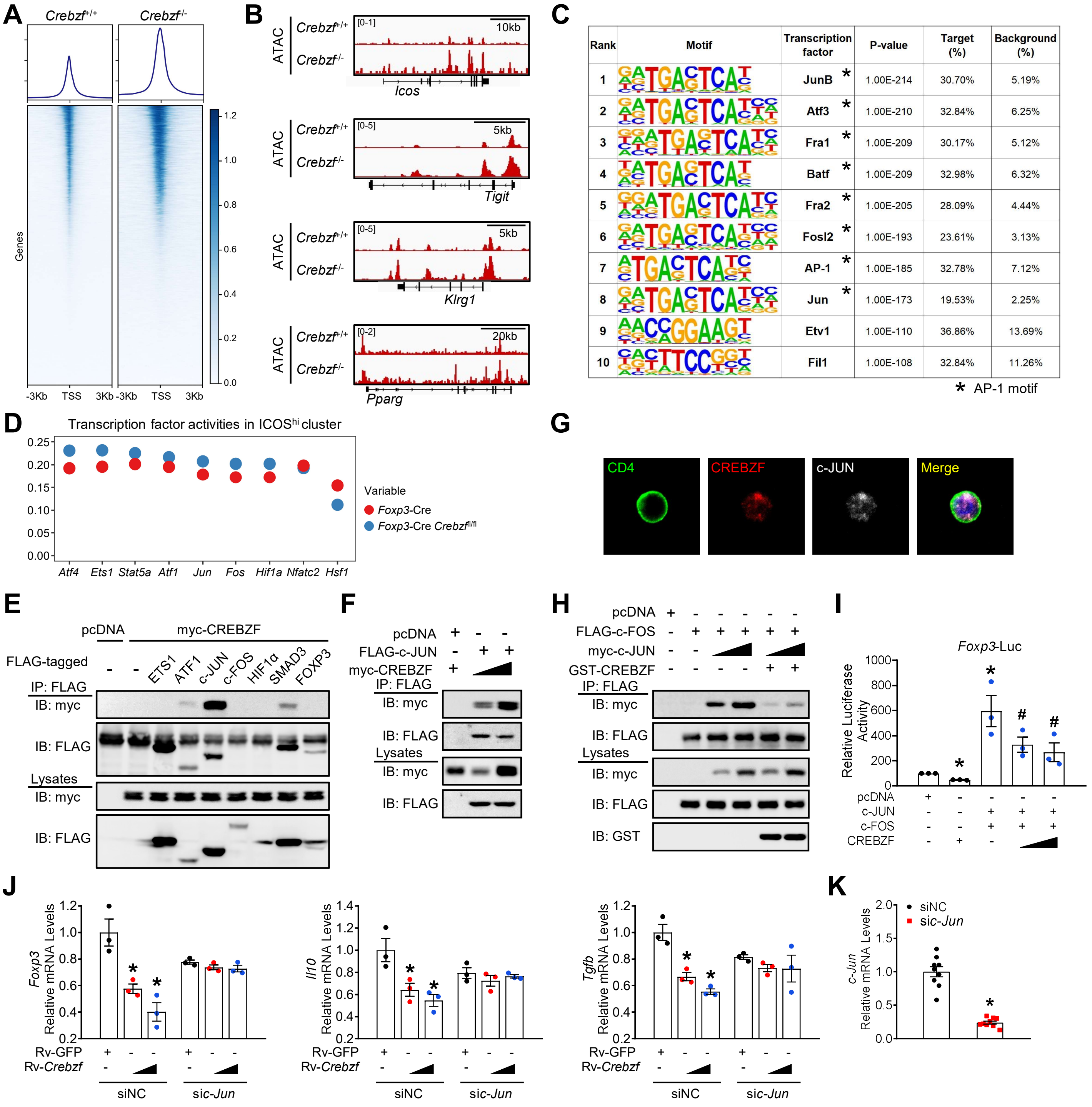

Rank	Motif	Transcription factor	P-value	Target (%)	Background (%)
1		JunB *	1.00E-214	30.70%	5.19%
2		Atf3 *	1.00E-210	32.84%	6.25%
3		Fra1 *	1.00E-209	30.17%	5.12%
4		Batf *	1.00E-209	32.98%	6.32%
5		Fra2 *	1.00E-205	28.09%	4.44%
6		Fosl2 *	1.00E-193	23.61%	3.13%
7		AP-1 *	1.00E-185	32.78%	7.12%
8		Jun *	1.00E-173	19.53%	2.25%
9		Etv1	1.00E-110	36.86%	13.69%
10		Fil1	1.00E-108	32.84%	11.26%

Supplementary Fig. S10 **Su W, Liu Y, Yan X, et al.**

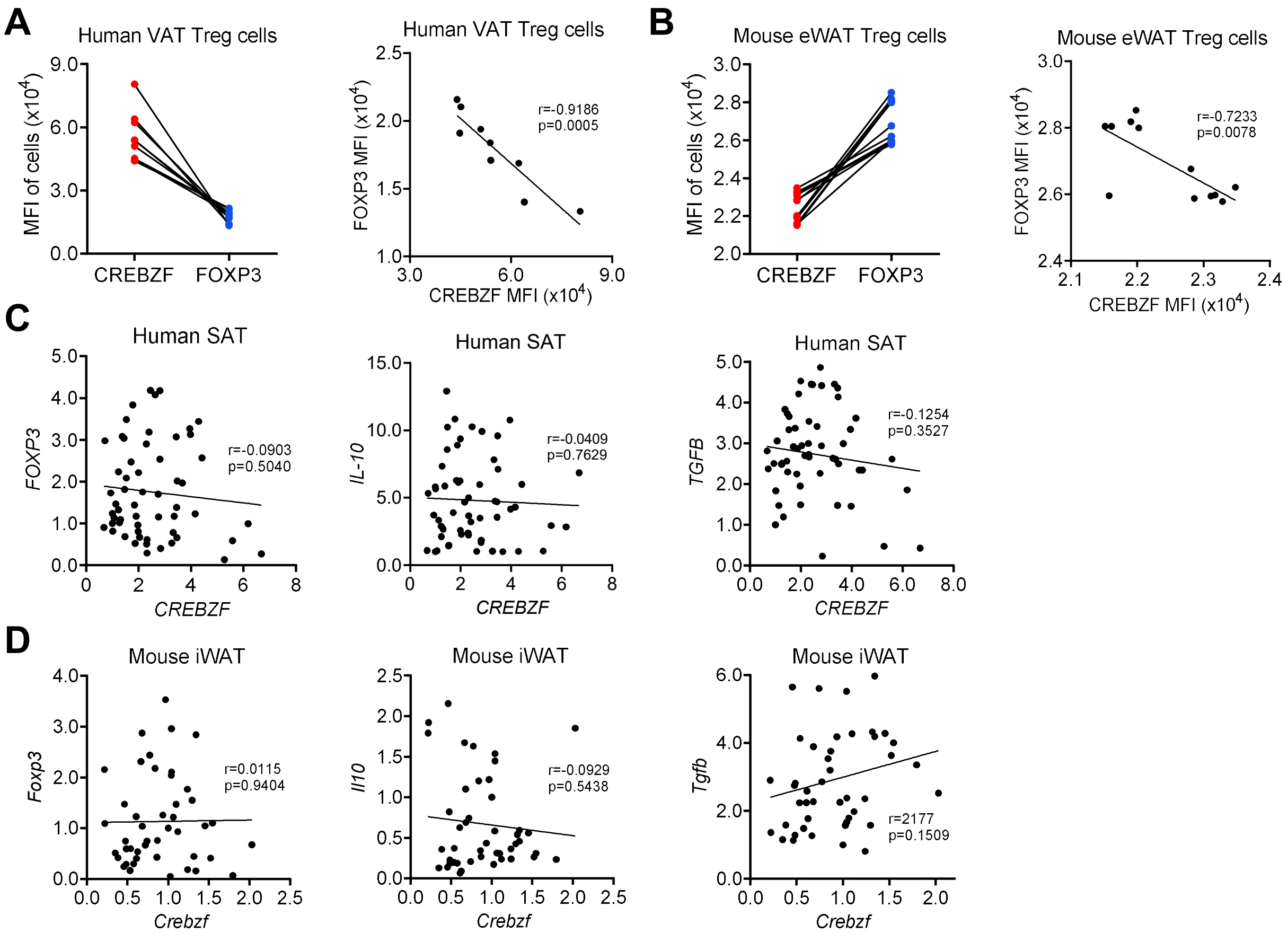

Table S1. Demographic information of human subjects

ID	Sex	Age (years)	BMI (kg/m2)
1	F	48	43.1
2	F	21	43.3
3	F	20	40.4
4	F	31	43.8
5	M	46	39.9
6	F	23	43.5
7	M	27	36.2
8	F	20	40.6
9	M	24	43.9
10	F	32	37.5
11	M	27	35.5
12	F	32	32.8
13	M	28	31.7
14	M	37	39.7
15	F	67	45.3
16	M	38	54.9
17	M	26	44.1
18	F	25	43.0
19	M	29	34.3
20	F	24	44.3
21	F	34	31.2
22	F	60	29.9
23	M	59	23.4
24	M	58	25.7
25	F	55	23.0
26	F	72	22.7
27	M	74	26.0
28	M	41	28.4
29	M	42	25.8
30	M	57	17.1
31	M	52	21.6
32	F	56	25.5
33	F	71	25.3
34	F	20	16.1
35	F	59	35.4
36	M	28	48.9
37	M	20	39.9

38	F	28	38.0
39	M	23	44.8
40	F	21	36.6
41	M	26	49.2
42	F	19	40.1
43	F	21	62.2
44	M	35	45.2
45	F	23	40.6
46	F	26	38.8
47	F	59	46.1
48	F	51	35.3
49	M	30	38.2
50	M	30	44.2
51	F	59	31.3
52	F	24	39.1
53	F	24	44.2
54	F	23	48.3
55	M	28	41.5
56	F	33	33.7
57	M	37	43.8
58	F	25	49.0
59	M	21	40.1
60	F	24	31.9
61	F	26	39.5
62	F	33	40.0
63	F	34	37.0
64	F	27	37.3
65	F	33	36.5
66	F	25	41.5
67	M	26	63.1
68	F	59	48.6
69	M	29	40.5
70	F	48	37.8
71	F	44	47.5
72	F	59	36.7
73	M	35	37.9
74	F	27	48.9
75	F	30	29.8
76	F	36	32.6

77	M	20	47.7
78	F	19	47.6
79	F	31	43.8
80	F	24	54.9
81	F	36	39.6
82	M	26	57.6
83	F	24	48.5
84	M	47	36.8
85	M	35	31.0
86	F	29	35.3
87	M	28	43.1
88	F	24	32.8
89	F	29	41.9

Table S2. Quantitative RT-PCR primers

Gene	Species	Forward primer	Reverse primer
Actb	mouse	CCACAGCTGAGAGGGAAATC	AAGGAAGGCTGGAAAAGAGC
Crebzf	mouse	AAGGTGTCGGTGGAGTTCTG	GGAGGAGAAAGGGGTAAACG
Ifng	mouse	CAGCAACAGCAAGGCGAAAAAGG	TTTCCGCTTCCTGAGGCTGGAT
Il1b	mouse	TTCGTGAATGAGCAGACAGC	GGTTTCTTGTGACCCTGAGC
Il6	mouse	AGTTGCCTTCTTGGGACTGA	TCCACGATTTCCCAGAGAAC
Tnfa	mouse	CGTCAGCCGATTTGCTATCT	CGGACTCCGCAAAGTCTAAG
Il17a	mouse	CAGACTACCTCAACCGTTCCAC	TCCAGCTTTCCCTCCGCATTGA
Il10	mouse	CGGGAAGACAATAACTGCACCC	CGGTTAGCAGTATGTTGTCCAGC
Il4	mouse	ATCATCGGCATTTTGAACGAGGTC	ACCTTGGAAGCCCTACAGACGA
Foxp3	mouse	CCTGGTTGTGAGAAGGTCTTCG	TGCTCCAGAGACTGCACCACTT
Aco2	mouse	GCCCAGATGGCTATGCTACAG	CGCAGGTCTTTCTCACCCC
Atp5a1	mouse	TCTCCATGCCTCTAACACTCG	CCAGGTCAACAGACGTGTCAG
Sdhb	mouse	CTGAATAAGTGCGGACCTATGG	AGTATTGCCTCCGTTGATGTTC
Atgl	mouse	CTTGAGCAGCTAGAACAATG	GGACACCTCAATAATGTTGGC
Hsl	mouse	GCTGGAGGAGTGTTTTTTTGC	AGTTGAACCAAGCAGGTCACA
Ppara	mouse	TACTGCCGTTTTCACAAGTGC	AGGTCGTGTTCACAGGTAAGA
Cpt1a	mouse	AGATCAATCGGACCCTAGACAC	CAGCGAGTAGCGCATAGTCA
Cpt2	mouse	CAGCACAGCATCGTACCCA	TCCCAATGCCGTTCTCAAAAT
Mcad	mouse	ATGCCTGTGATTCTTGCTGGA	ACATCTTCTGGCCGTTGATAAC
Acox1	mouse	CCGCCACCTTCAATCCAGAG	CAAGTTCTCGATTTCTCGACGG
Cd36	mouse	GGACATTGAGATTCTTTTCCTCTG	GCAAAGGCATTGGCTGGAAGAAC
Icos	mouse	GCAGCTTTCGTTGTGGTACTCC	TGTGTTGACTGCCGCCATGAAC
Cd3e	mouse	CTCAGAAGCATGATAAGCAC	CAGAGTGATACAGATGTCAAC
ACTB	human	GATGAGATTGGCATGGCTTT	GTCACCTTCACCGTTCCAGT
CREBZF	human	AGAAGGAGTACGTGATGGGG	CGTAGGTAGCGACTCTCCTC
CD3	human	GCATTTTCGTCCTTGCTGTTGGG	GGTCATCTTCTCGATCCTTGAGG
CD4	human	CCTCCTGCTTTTCATTGGGCTAG	TGAGGACACTGGCAGGTCTTCT
CD8	human	ACTTGTGGGGTCCTTCTCCTGT	TGTCTCCCGATTTGACCACAGG
FOXP3	human	GGCACAATGTCTCCTCCAGAGA	CAGATGAAGCCTTGGTCAGTGC
TGFB	human	TACCTGAACCCGTGTTGCTCTC	GTTGCTGAGGTATCGCCAGGAA
IL-10	human	TCTCCGAGATGCCTTCAGCAGA	TCAGACAAGGCTTGGCAACCCA

Supplemental Experimental Procedures

Reagents	Company	Catalog number
Recombinant Mouse IL-2	Novoprotein	CK24
Recombinant Mouse IL-4 (C-6His)	Novoprotein	CK74
Recombinant Mouse IL-6	Novoprotein	CG39
Recombinant Mouse IL-12	Novoprotein	CM39
Recombinant Mouse/Rat TGF-beta 1	Novoprotein	CK33
Collagenase	Sigma	C2139-100MG
Collagenase, Type 1 (CLS-1),1 gm	worthington	LS004197
Deoxyribonuclease I from bovine pancreas (DNase I)	Sigma	DN25-100MG
phorbol 12-myristate 13-acetate, PMA	Sigma	P1585
Ionomycin	Sigma	407952
Palmitic acid	Sigma	P0500-10G
Ethyl palmitate	MedChemExpress	HY-N2086
Lecithin	MedChemExpress	HY-B2235
Glycerol	MedChemExpress	HY-B1659
Insulin (human) recombinant	TOCRIS Bioscience	3435
D-Glucose	Sigma	G8270-100G
Sodium L-lactate	sigma	71718-10G
TRIzol Reagent	Life Technologies	15596026CN
Foxp3 Transcription Factor Staining Buffer Set	eBioscience	00-5523-00
Fixation/Permeabilization Solution Kit with BD GolgiStop	BD	554715
Rat/Mouse insulin ELISA kit	Millipore	E2RMI-13K
Anti-Mouse IL-10 Antibody	MedChemExpress	HY-P990001
Rat IgG1 kappa, Isotype Control	MedChemExpress	HY-P99979
HiScript III RT SuperMix for qPCR (+gDNA wiper)	Vazyme	R323-01
ChamQ SYBR qPCR Master Mix (High ROX Premixed)	Vazyme	Q341-02
TruePrep DNA Library Prep Kit V2 for Illumina	vazyme	TD501
TruePrep Index Kit V2 for Illumina	vazyme	TD202
VAHTS DNA Clean Beads	vazyme	N411-01
VAHTS DNA Adapters set1 for Illumina	vazyme	N801-01
HiScript III RT SuperMix for qPCR (+gDNA wiper)	vazyme	R323-01
ChamQ SYBR qPCR Master Mix (High ROX Premixed)	vazyme	Q341-03

Antibody (Clone number)	Company	Catalog number
Anti-CD45-APC-Cy7 (clone 30-F11)	BD biosciences	557659
Anti-CD3e-FITC (clone 145-2C11)	BD biosciences	553061
anti-CD3e-FITC (clone 145-2C11)	Biolegend	100306

Anti-CD3e-PE-Cy7 (clone 17A2)	Biolegend	100220
Anti-CD4-BV421 (clone GK1.5)	BD biosciences	562891
Anti-CD4-APC (clone RM4-5)	BD biosciences	553051
Anti-CD4-BV421 (clone BM8)	Biolegend	123132
Anti-CD4-BV421 (clone GK1.5)	Biolegend	100438
Anti-CD8a-BV650 (clone 53-6.7)	Biolegend	100742
Anti-CD8a-PE (clone 53-6.7)	BD biosciences	553032
Anti-CD44-APC (clone IM7)	Biolegend	103012
Anti-CD44-FITC (clone IM7)	eBioscience	11-0441-82
Anti-CD62L-PE (clone MEL-14)	eBioscience	12-0621-82
Anti-IFNγ-PE-Cy7 (clone XMG1.2)	eBioscience	25-7311-82
Anti-IL-4-PE (Clone 11B11)	BD biosciences	554435
Anti-IL-17A-Alexa Fluor 647 (clone TC11-18H10.1)	Biolegend	506912
Anti-RORγT-PE (clone B2D)	eBioscience	12-6981-80
Anti-T-bet-PE-Cy7 (clone eBio4B10 (4B10))	eBioscience	25-5825-80
Anti-CD25-PE (clone PC61.5)	eBioscience	12-0251-82
Anti-Foxp3-APC (clone FJK-16s)	eBioscience	12-5773-82
Anti-Foxp3 eFluor 450 (clone FJK-16s)	eBioscience	48-5773-82
Anti-IL-10-PE (clone JES5-16E3)	Biolegend	505007
Anti-TGF-β1-APC (clone TW7-16B4)	Biolegend	141405
Anti-KLRG1-PE (clone 2F1)	Biolegend	138407
Anti-ICOS-APC (clone 7E.17G9)	Biolegend	117419
Fixable Viability Dye eFluor 506	eBioscience	65-0866-18
Fixable Viability Dye eFluor 780	eBioscience	65-0865-18
Anti-CREBZF (Polyclonal)	Abclonal	A3477
Anti-Zhangfei antibody (Polyclonal)	Abcam	ab28700
Anti-Zhangfei antibody (Polyclonal)	Abcam	ab228834
Anti-β-actin (AC-15)	Santa Cruz	sc-69879
Anti-CD3 (145-2C11)	bioxcell	BE0001-1
Anti-CD28 (37.51)	bioxcell	BE0015-1
Alexa Fluor 488 goat anti-rabbit IgG (Polyclonal)	Invitrogen	A-11034
Alexa Fluor 594 goat anti-rabbit IgG (Polyclonal)	Invitrogen	A-11012
Alexa Fluor 488 goat anti-mouse IgG (Polyclonal)	Invitrogen	A-11001
Alexa Fluor 594 goat anti-mouse IgG (Polyclonal)	Invitrogen	A-11005

Mouse model. The genotyping primers for detecting the mice expressing *Cd4*-Cre recombinase were: ATCTGGCATTTCTGGGGATTG (forward) and GGCAACACCATTTTTTCTGACC (reverse). Two pairs of primers for detecting the mice expressing *Foxp3*-Cre recombinase were: CTATGGAAACCGGGCGATGA (forward) and AGTGGCAAGTGAGACGTGGG (reverse) for *Foxp3*-WT alleles; AGGATGTGAGGGACTACCTCCTGTA (forward) and TCCTTCACTCTGATTCTGGCAATTT (reverse) for *Foxp3*-YFP alleles. Two pairs of primers for detecting *Crebzf* alleles: GCTTGCAGTTTAGAGAGAAACAGC (forward) and CAGCCAGAGTATCGCGAGATTC (reverse) for detecting wild-type *Crebzf* alleles; TTGACAATAAGTATTGAGGCATGCG (forward) and TTTCCAACTTCTCAAGTGGTGAAC (reverse) for detecting the *Crebzf* alleles lacking excised floxed fragment.

***In vivo* palmitic acid treatment.** Palmitic acid for *in vivo* treatment was performed as previously described (1). In brief, the mice were intraperitoneally administered 3 μmol/g body weight emulsified ethyl palmitate or vehicle daily for 3 weeks. Ethyl palmitate was dissolved with lecithin and glycerol in water to produce a mixture and emulsified using a homogenizer. The solution containing 1.2% lecithin and 2.5% glycerol alone was used as the vehicle control. Adipose tissue and spleen from mice were harvested for flow cytometry analysis.

Plasmids. The plasmid encoding FLAG-tagged c-JUN, FLAG-tagged c-FOS, FLAG-tagged ATF1, FLAG-tagged SMAD3, FLAG-tagged HIF1α, FLAG-tagged ETS1 and myc-tagged c-JUN were constructed by cloning complementary DNA (cDNA) as indicated into the sites of pcDNA3.1 or pCDH vectors. The pGL3-*Foxp3*-promoter plasmid encoding a luciferase transgene regulated by the *Foxp3* promoter was constructed by cloning *Foxp3* promoter into a pGL3-based backbone. The retrovirus plasmid (MSCV-IRES-*Crebzf*) was constructed by cloning cDNA encoding CREBZF into a retroviral expression vector (MSCV-IRES-GFP), which also encodes an IRES-GFP cassette. Plasmids were transferred by PEI or lipofectamine 2000 (Invitrogen), and the medium was changed after 8 h. Cells were treated indicated after 24-36 hours and analyzed.

Immunoblots and immunoprecipitation analysis. Immunoblotting analysis was carried out as described previously (2, 3). Tissues and cells were lysed in NP-40 lysis buffer (50mM Tris-HCl pH 8.0, 150mM NaCl, 1% NP-40, 5 mM EDTA, 1 mM EGTA, 1 mM sodium orthovanadate, 10 mM sodium

fluoride, 1 mM phenylmethylsulfonyl fluoride, 2 μg/ml aprotinin, 5 μg/ml leupeptin, and 1 μg/ml pepstatin). Lysates were centrifuged, and the supernatant was detected protein concentration by BCA. For immunoblotting, total protein (10-50 μg per lane) was separated by 10% sodium dodecyl sulfate-polyacrylamide gel electrophoresis (SDS–PAGE) and transferred to polyvinylidene difluoride (PVDF) membranes in a transfer buffer consisting of 25 mM Tris base, 190 mM glycine, and 20% methanol. The membranes were blocked by 5% non-fat milk in Tris-buffered saline with 0.1% Tween 20 (TBST) and incubated with primary antibody followed by horseradish peroxidase-conjugated secondary antibody. For immunoprecipitation, cell lysates containing overexpressed recombinant or endogenous proteins were incubated with specific primary antibodies overnight, and protein A/G Sepharose beads were added for another 4 hours at 4ºC. The precipitates were washed three times with ice-cold lysis buffer and analyzed by immunoblots.

Retroviral package and infection. Retrovirus plasmid (MSCV-IRES-*Crebzf*) and pCL-ECO (packaging plasmid) were transfected into HEK293T cells using polyethylenimine (PEI), retrovirus supernatant was harvested 48 h after transfection (4). Naïve $CD4^+$ $CD25^-$ $CD62L^{hi}CD44^{low}$ T cells form spleen of mice were FACS sorted and stimulated with the plate-bound anti-CD3 (5 mg/mL) and anti-CD28 (2 mg/mL) under neutral conditions (anti-IFNγ (10 mg/mL) and anti-IL4 (10 mg/mL)) for 20 h, then infected with retrovirus in the presence of polybrene (8 mg/mL) with centrifugation (1800rpm, 32 degree) for 90 min, and fresh medium was changed 4 h after infection.

Small interfering RNA (siRNA) knockdown. Knockdown experiments of c-JUN in mouse Treg cells were performed using siRNA oligonucleotides from GenePharma (Shanghai, China). The sequences of the siRNA oligos are as follows: si*c-Jun* CAGCAAUGGGCACAUCACCACUACA (sense), UGUAGUGGUGAUGUGCCCAUUGCUG (anti-sense); Negative control siRNA (siNC), UUCUCCGAACGUGUCACGU (sense), ACGUGACACGUUCGGAGAATT (anti-sense). Cells were transfected with siRNAs using Hieff Trans TM in virto siRNA/miRNA transfection regent (cat. 40806ES, YEASEN).

Dual luciferase activity assays. HEK293T cells were co-transfected with 0.5 μg of empty vector or plasmids encoding c-JUN, c-FOS and CREBZF as indicated, along with 0.5 μg luciferase reporter plasmids (pGL3-*Foxp3*-Luc) and 80 ng of Renilla luciferase plasmid pRL-SV40 (Promega) as an

internal control in 12 well plates. 24 hours post transfection, the media was changed, and the cells were allowed to recover for additional 18 hours. Dual luciferase assays for Firefly luciferase and Renilla luciferase activities were performed in duplicates according to the manufacturer's protocols (Promega). Luciferase activity was measured using an Infinite 2000pro plate reader (TECAN, Switzerland). The Firefly luciferase activity was normalized to the Renilla luciferase activity (Firefly luciferase/Renilla luciferase) and presented as relative luciferase activity.

ATAC-seq. ATAC-seq was performed as previously described (5). 5×10^4 Treg cells were isolated from the eWAT of mice fed with HFHS diet for 16 weeks. The unfixed nuclei of these cells were tagged using tn5 transposase (TruePrep DNA Library Prep Kit V2 for Illumina, Vazyme) for 30 min at 37 °C, and the resulting library fragments were generated by 13 PCR cycles, and finally sequenced on an Illumina HiseqXten-PE150. Sequence tags from ATAC-seq libraries were aligned to the mm10 using the BWA aligner (Version 0.7.17-r1188). Postalignment processing of reads was performed with Samtools (Version 1.16.1) and Bedtools (Version 2.30.0) by removing duplicate reads and selecting for high quality reads. Heatmap and visualization of peak distribution along genomic regions of interested genes were presented by deepTools (Version 3.5.1) and Integrative Genomics Viewer (IGV) software (6). Peak calling was identified using MACS (Version 2.2.7.1) and then annotated by ChIPseeker (Version 1.38.0). Furthermore, EdgeR (Version 4.0.2) was used to detect differentially accessible regions (DARs) ($|\log2FC| > 0.75$, adjusted P value < 0.05) between CREBZF$^{+/+}$ and CREBZF$^{-/-}$ groups. To identification putative regulators, Homer (Version 4.9.1) was used to perform motif enrichment of the DARs. ClusterProfiler (Version 4.10.0) was used to perform enrichment analysis of the genes nearby DARs to GO, KEGG and Reactome pathway.

Reference

1. Ogawa Y, Imajo K, Honda Y, Kessoku T, Tomeno W, Kato S, et al. Palmitate-induced lipotoxicity is crucial for the pathogenesis of nonalcoholic fatty liver disease in cooperation with gut-derived endotoxin. *Sci Rep.* 2018;8(1):11365.
2. Li Y, Xu S, Mihaylova MM, Zheng B, Hou X, Jiang B, et al. AMPK phosphorylates and inhibits SREBP activity to attenuate hepatic steatosis and atherosclerosis in diet-induced insulin-resistant mice. *Cell Metab.* 2011;13(4):376-88.
3. Gong Q, Hu Z, Zhang F, Cui A, Chen X, Jiang H, et al. Fibroblast growth factor 21 improves hepatic insulin sensitivity by inhibiting mammalian target of rapamycin complex 1 in mice. *Hepatology.* 2016;64(2):425-38.
4. Qu J, and Yang Z. Protocol to produce high-titer retrovirus for transduction of mouse bone marrow cells. *STAR Protoc.* 2021;2(2):100459.
5. Buenrostro JD, Wu B, Chang HY, and Greenleaf WJ. ATAC-seq: A Method for Assaying Chromatin Accessibility Genome-Wide. *Curr Protoc Mol Biol.* 2015;109:21 9 1- 9 9.
6. Robinson JT, Thorvaldsdottir H, Winckler W, Guttman M, Lander ES, Getz G, et al. Integrative genomics viewer. *Nat Biotechnol.* 2011;29(1):24-6.
