# Supplementary material for: Palmitic acid coordinates impaired visceral adipose ICOShi Treg-mediated immunosuppression and systemic metabolic disturbance during obesity
Source: J Clin Invest. 2026 Jul 23;136(18):e207089. doi: 10.1172/JCI207089 (PMC13574151; doi:10.1172/JCI207089)

Full unedited blot/gel for Figure S2C

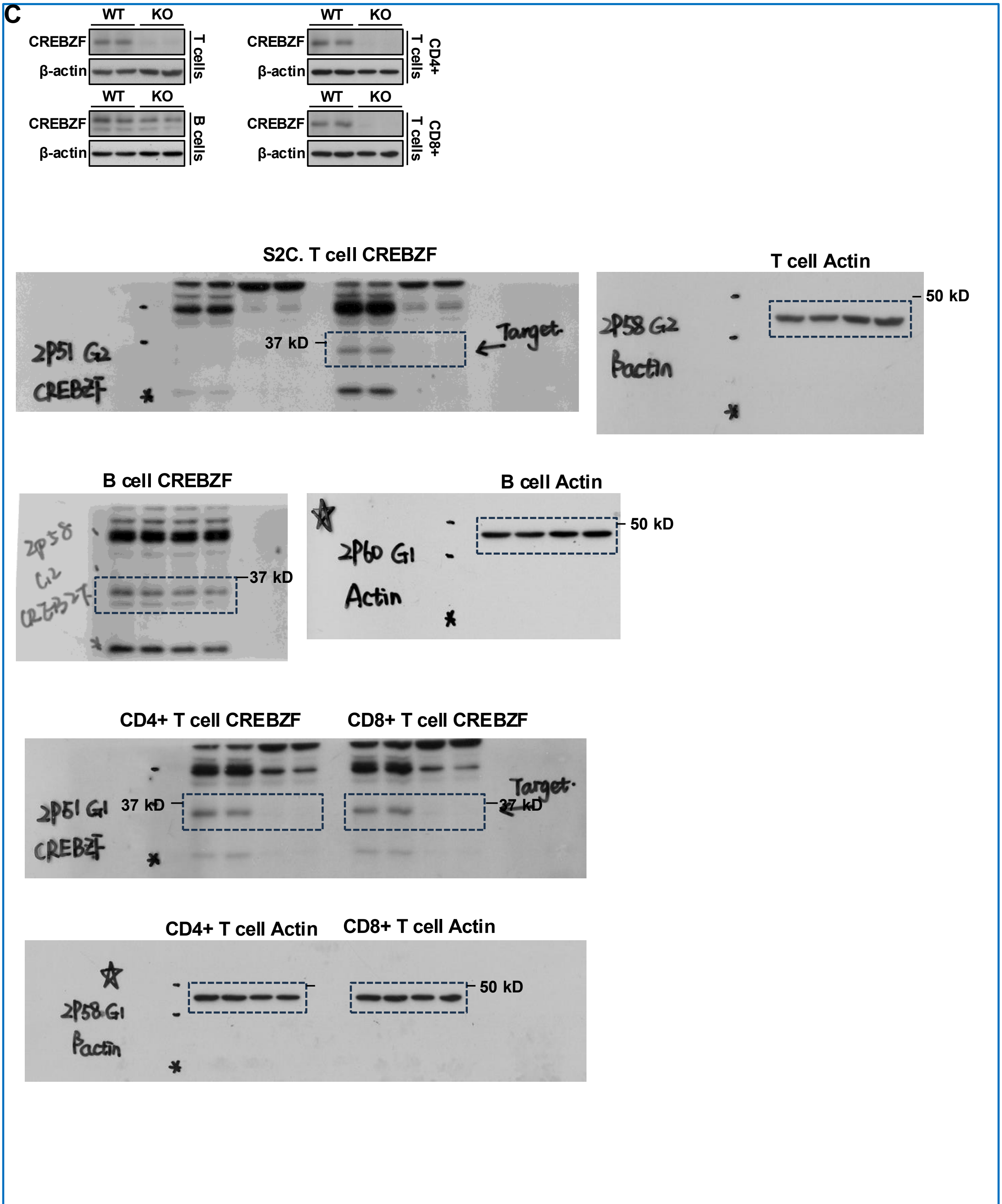

Full unedited blot for Figure S9B, 9C, and 9E

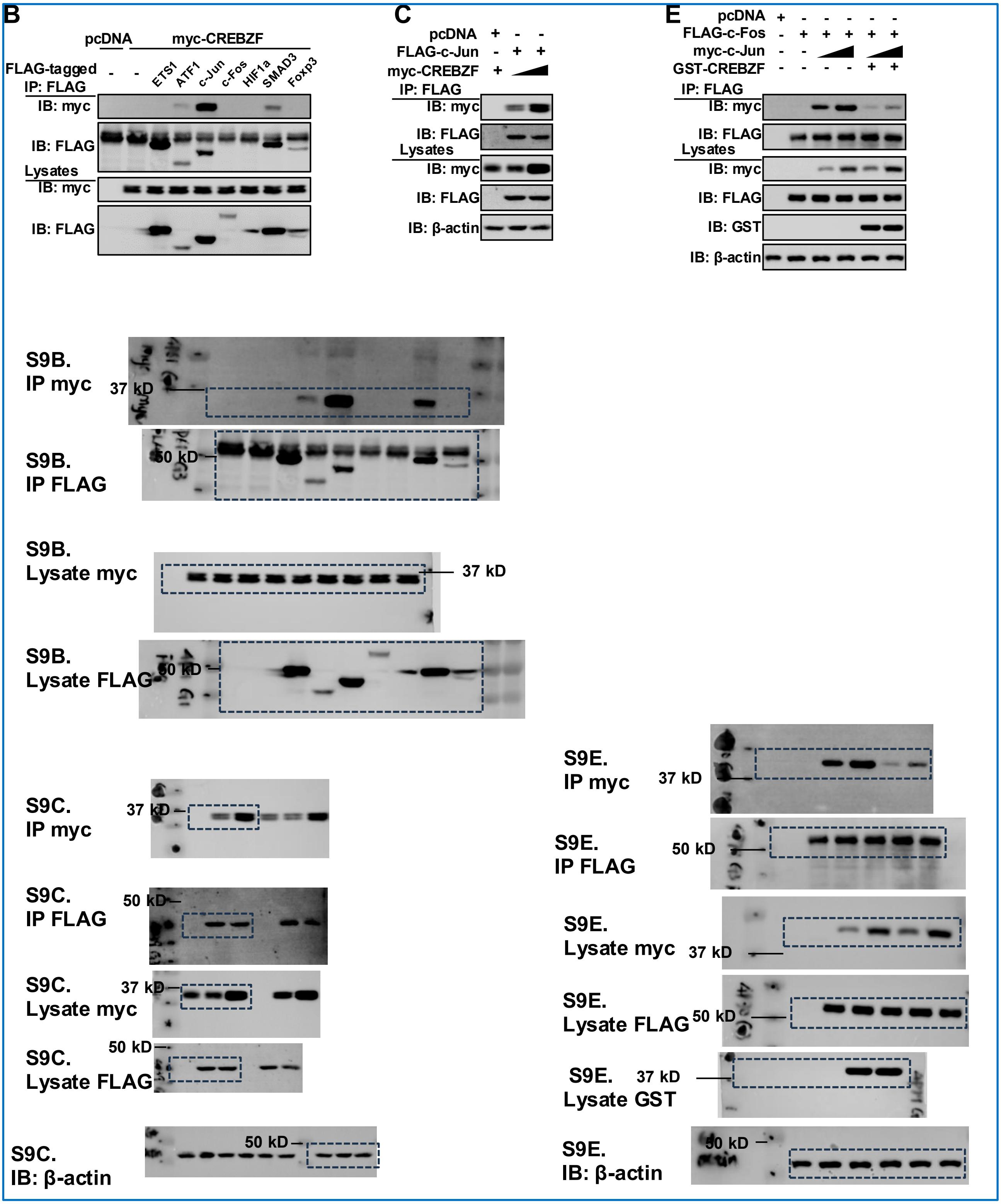

Supplement: Supplementary file 2 [file jci-136-207089-s043.pdf]
